# Secure Multiplicative Aggregation and Key-Reuse Optimization: Achieving Dropout Resilience with Amortized Efficiency

**DOI:** 10.3390/e28030358

**Published:** 2026-03-22

**Authors:** Hongyuan Cai, Bei Liang, Yue Qin, Jintai Ding

**Affiliations:** 1 Department of Mathematical Sciences, Tsinghua University, Beijing 100084, China; chy22@mails.tsinghua.edu.cn; 2 Beijing Institute of Mathematical Sciences and Applications, Beijing 101408, China; yueqin@bimsa.cn; 3 School of Cyber Science and Technology, Beihang University, Beijing 100191, China; 4 School of Mathematics and Physics, Xi’an Jiaotong-Liverpool University, Suzhou 215123, China; jintai.ding@gmail.com

**Keywords:** secure aggregation, privacy, key reuse

## Abstract

We present the first secure multiplicative aggregation protocol as a variant of secure aggregation. In this case, a server can compute the component-wise product of the input vectors of users while handling the possible dropout of users during protocol execution. Using pairwise masks, threshold secret sharing and the secure aggregation protocol itself, our construction is correct and secure against semi-honest adversaries. We also consider secure aggregation protocols for the case in which fixed users can reuse their private keys to do aggregation many times, and we propose key reusable secure aggregation protocols. Our protocols have an overhead polynomial in the number of users. We conduct a comprehensive evaluation of our proposed protocols. For multiplicative aggregation protocol, experiments varying the number of users (*K*) from 50 to 300 (with fixed input size $X_{u}=100$ KB) demonstrate that user computation scales monotonically with *K* and is largely insensitive to dropout rates. In contrast, server computation is highly dropout-sensitive and exhibits a steeper growth rate with respect to *K*. When varying the input size (10–250 KB) with a fixed *K*, both user and server communication overheads increase linearly, while server computation remains the primary bottleneck affected by dropouts. We compare reusable and non-reusable secure aggregation protocol over repeated interactions q∈{1,…,10} at $X_{u}=100$ KB and K=100, showing that reusing Round 1 reduces the cumulative user computation time by about 2.5 times and reduces the cumulative server computation overhead by about 1.2 times at q=10 while leaving the server communication overhead nearly unchanged, which indicates that the overall communication overhead is dominated by the non-reused rounds.

## 1. Introduction

Secure aggregation is a kind of specific secure multiparty computation, which enables a server to interact with a number of parties who each have a private information vector and finally output the sum of these parties’ private vector while, during the process, no private information is revealed except for the output. Secure aggregation can be used in the federated learning setting of a machine learning model [1], wherein each user maintains the private database locally on his device, the server computes the sum of users’ local updates by secure aggregation to get an updated global model and pushes back the global update to realize updates for each user.

Secure aggregation has been widely studied in recent years, various cryptographic approaches have been proposed to solve it, and its different model variants have been discussed. Major approaches are multiparty computation (MPC), homomorphic encryption (HE) and differential privacy (DP).

MPC-based techniques for secure aggregation mainly utilize Yao’s garbled circuit [2], homomorphic encryption or secret sharing [3]. Generic secure multiparty computation is in such a setting, where parties with private inputs want to collaborate with each other to compute some functionality of their inputs, while keeping their inputs unknown to others. The fundamental results of MPC include BGW protocol [4], Yao’s garbled circuit [2], the GMW protocol [5], the SPDZ protocol [6], homomorphic encryption-based tiny OT protocol [7], BMR protocol [8], etc. When applying generic secure multiparty computation protocols to solve secure aggregation, all that is needed is to take the functionality as a summation function; thus, it is also easy to solve different kinds of variants of secure aggregation (such as computing a weighted sum); however, these protocols commonly have high communication costs, and communication-efficient variants require extensive offline computation [9], especially when the number of users is large and private vectors are high-dimensional. Additionally, these protocols cannot handle the possible dropout of users during the execution.

Secure aggregation protocols based on threshold homomorphic encryption systems (such as Paillier encryption system) allow aggregation to be performed on encrypted information and can solve the secure aggregation variant that users may dropout at any time of the protocol execution. Joye et al. have proposed a HE-based secure aggregation protocol [10], but it is in need of a trusted server to distribute and update keys. Leontiadis et al. have done some improvement to it [11], so that users are able to join in and dropout without having to update keys. Halevi, Lindell and Pinkas presented a protocol [12] to securely compute the sum in one round of interaction between the server and each user using homomorphic encryption. They require the communication between user and server to be carried out sequentially (thus no need for users to be online simultaneously) and cannot handle a dropout case. The privacy security guarantees of HE-based protocols depend on the size of the encrypted data, so the computation cost in the encrypted domain could be very expensive [13], and computation-efficient variants always need additional trust assumption, such as the existence of a trusted third party.

Secure aggregation protocols based on differential privacy (DP), which is a noise release mechanism, add virtual noise to each user’s private information locally to protect privacy. Differential privacy makes sure that the removal of any single element from the database will not affect the computation output significantly; as such, the output cannot be used to infer any single private information [14]. In the context of federated learning, users add artificial noise to their own local updates, so that any local update will not be identified by the server [15,16,17]. Shi et al. presented a secure aggregation protocol based on differential privacy and homomorphic encryption [18], and they have given a rigorous analysis of distributed noise generation. Chan et al. extend the result to provide robustness against user dropouts [19]. The DP approach entails a trade-off between convergence performance and privacy protection, as stronger privacy guarantees lead to a degradation in the convergence performance.

Moreover, there are secure aggregation protocols based on Dining Cryptographers Networks. DC-net is a type of communication network that provides anonymity by using pairwise masking of inputs. One user sends one anonymous message at a time that can be viewed as the restricted case of secure aggregation. Corrigan et al. increased the efficiency of DC-net protocols [20], but it is not robust to user dropout and involves expensive overhead [21].

Pairwise additive masking can also be used to construct a secure aggregation protocol. In the work of Asc and Castelluccia [22], pairs of users use a Diffie–Hellman key exchange [23] to agree on pairwise masks; each user computes the sum of their private data, pairwise masks and a self-mask and then sends it to the server. As the summation of the additive masks will cancel out, the server can sum up the masked data it has received to obtain the result. If some users drop out, the server asks the remaining users to send the sum of the pairwise masks associated with the dropped users, added to their self-mask, and thereby subtracts them to obtain the correct sum. The protocol needs only constant rounds of interaction, but the communication overhead of the recovery phase is the limitation; also, if additional users drop out during this phase, the protocol has to abort. A protocol more robust to users’ dropout is proposed by Bonawitz et al. [24] that can efficiently compute the sum of high-dimensional vectors from a large number of users. The protocol makes use of threshold secret sharing; thus, when reconstructing masks in the recovery phase, the server has an overhead quadratic to the number of users. So et el. have described a protocol robust to users dropout that users interact with, server group by group [25]; the overhead is poly-logarithmic to the number of users, but the number of interaction rounds turns into $\frac{n}{\log n}$. By changing the complete communication graph in the protocol of Bonawitz et al. [24] to a *k*-regular graph, Bell et al. [26] realized a secure aggregation protocol with a poly-logarithm overhead and constant rounds of interaction, which is also robust to user dropout.

Due to the extensive research on secure aggregation, it is natural to think about a variant of secure aggregation, secure multiplicative aggregation. We consider a model similar to the protocol of Bonawitz et al. [24], in which multiple users, each holding a high-dimensional private vector, interact with a central server, such that the server finally obtains the component-wise product of input vectors. The users cannot communicate with each other directly and may drop out at any time during the protocol execution. Once a user has dropped out, they cannot go back to the protocol again. The server knows the identity of dropout users, and the output of the server is the component-wise product of private input vectors of some users who have not dropped out before sending the message related to their private vector to the server. In this paper, we present a secure multiplicative aggregation protocol. We point out that the main challenge of constructing a secure multiplicative aggregation protocol is dealing with the possible 0 components in input vectors, and we introduce auxiliary vectors to solve the problem.

Moreover, in the scenarios of federated learning [1], multiple rounds of training are needed until the model converges. Therefore, we consider a multi-time setting where the set of initial users is fixed, each user has some private inputs, and they are willing to perform secure additive/multiplicative aggregation many times on different inputs. Utilizing secure additive aggregation protocols [24,26] or the secure multiplicative protocol that we have proposed multiple times is a natural way to solve the problem. However, it turns out that the public–private key pairs of users are unreusable in these protocols for security concerns since the server may obtain some users’ private keys in the protocol execution. In this paper, we construct additive/multiplicative aggregation protocols that allow users to reuse their public–private key pairs during multiple executions, so as to reduce costs.

### Our Main Contribution

Our contribution to secure aggregation mainly contains:

(1) Based on a practical secure aggregation protocol [24], we propose a secure multiplicative aggregation protocol. The protocol defines the interaction between a server and *n* users who each have an *m*-dimension private vector with a component belonging to $Z_{R}$ as the input, and finally, the server outputs the component-wise product of online users’ private vector (on some finite field). The protocol allows users to drop out at any time, and once the number of online users is less than the secret sharing threshold parameter *t*, the protocol will abort with no output. When a server obtains the output, the protocol guarantees the correctness of the output with a probability of more than $1-\frac{1}{2^{\eta }}$, where η represents the correctness parameter. For privacy, our secure multiplicative aggregation protocol is secure against a semi-honest adversary who controls less than or equal to *t* parties (the parties can be users or the server), where *t* is the threshold parameter of secret sharing.

It can be noted that, when the input vectors have no 0 components, secure multiplicative aggregation can be reduced to secure additive aggregation by processing the components with a logarithm. Therefore, the main challenge is to deal with possible 0 components in input vectors. Our key observation is that, when *R* is a prime number, the indices of 0 components in input vectors fully determine the indices of 0 components in the output result of secure multiplicative aggregation. To leverage the property, we introduce auxiliary vectors that contain the information about the indices of 0 components in input vectors. A secure additive aggregation protocol is executed on auxiliary vectors to ensure that the server only learns information about the indices of 0 components in the output result of secure multiplicative aggregation; thus, security is guaranteed.

(2) We consider a case adapted to federated learning [1], where some fixed set of users want to perform secure additive/multiplicative aggregation many times. Based on the construction of Bonawitz et al. [24] and our secure multiplicative aggregation protocol, we propose a key reusable secure additive aggregation protocol and a key reusable secure multiplicative aggregation protocol.

We have observed that the reason why the public–private key pairs of users cannot be reused in the construction of Bonawitz et al. [24] and our secure multiplicative aggregation is that the server will reconstruct the private keys of some users, which are then used to compute the pairwise masks to be removed from the masked inputs. To maintain the privacy of the long-term private keys and make them reusable, we leverage one-time private keys for each aggregation execution and use a bilinear map, rather than key agreement, to compute pairwise masks while avoiding the appearance of one-time public keys.

(3) We implement secure multiplicative aggregation and reusable aggregation protocols and evaluate their performance. We benchmark a secure multiplicative aggregation protocol under different user sizes and different dropout rates to evaluate the computational and communication overhead for users and the server. When a user’s input is fixed at $X_{u}=100$ KB, with the number of users, *K*, increasing from 100 to 500, the average per-user computation time increases by about 14.2–14.5 times, while the total server computation time increases by about 27.7–69.5 times, depending on the dropout rate. When the dropout rate increases from 0% to 30% at K=500, the total server computation time increases by about 1.61 times, whereas the user’s computation time remains essentially unchanged. From the communication perspective, increasing the number of users, *K*, from 100 to 500 enlarges the average per-user communication overhead by about 3 times while leaving the total server communication overhead almost unaffected by the dropout rate.

For the reusable secure aggregation protocol, increasing the input size from 100 KB to 500 KB increases the communication overhead nearly proportionally, by about 4.8 times on the server side and about 1.5 times on the user side, while dropout impacts are mainly reflected in computation. Moreover, across repeated interactions, q∈{1,…,10}, reusing Round 1 yields clear computational savings while leaving the communication overhead nearly unchanged. At q=10, it reduces the cumulative user and server computation time by about 2.49 times and 1.18 times, respectively, indicating that reuse is particularly effective in amortizing user-side computation, whereas the overall communication cost remains dominated by the non-reused rounds.

## 2. Preliminaries

We introduce the main cryptographic primitives in this section and refer to Appendix A for the description of Authenticated Encryption (AE) and Pseudorandom Generator (PRG). We denote as λ and η the security and correctness parameters, respectively.

### 2.1. Secret Sharing

What we rely on is Shamir’s threshold *t* secret sharing [3], which allows a user to split a secret, *s*, into *n* parts, such that any more than or equal to *t* shares can be used to reconstruct *s*, while any less than or equal to t−1 shares give no information about *s*.

Shamir’s secret sharing scheme is defined over a finite field, F; for the sake of security, the size of field F should satisfy $l>2^{\lambda }$ (here, λ is a security parameter), so we might take $F=Z_{p}$, where $p>2^{\lambda }$ is a public big prime number. The scheme is performed between a dealer and *n* users; assume that *n* users can be identified with distinct field elements in F (it has an implicit requirement, p>n), and denote the set of identities of users as U⊆F. Given these parameters, Shamir’s *t*-out-of-*n* secret sharing scheme consists of two algorithms. The sharing algorithm $\mathrm{SS}.\mathrm{share}\left(s,t,U\right)\to {\left\{\left(u,s_{u}\right)\right\}}_{u\in U}$ takes as input a secret, s∈F, a set of users’ identities, *U*, and a threshold, t≤|U|; it outputs a set of shares, $s_{u}$, each of which is associated with a user, u∈U. The reconstruction algorithm $\mathrm{SS}.\mathrm{recon}({\left\{\left(u,s_{u}\right)\right\}}_{u\in V},t)\to s$ takes as input a subset, V⊆U, such that |V|≥t, the shares corresponding to subset *V* and threshold *t*; it outputs an element, *s*, in field F.

The above algorithms can be achieved using Lagrange interpolation. For the sharing algorithm $\mathrm{SS}.\mathrm{share}\left(s,t,U\right)\to {\left\{\left(u,s_{u}\right)\right\}}_{u\in U}$, the dealer chooses independently and uniformly distributed elements, $a_{1},\ldots ,a_{t-1}$, from F, defines a polynomial of degree t−1 in F[x] as $p_{s}\left(x\right)=s+a_{1}x+\ldots +a_{t-1}x^{t-1}$, and then takes $s_{u}=p_{s}\left(u\right)$ to be the share corresponding to user *u*. For the reconstruction algorithm $\mathrm{SS}.\mathrm{recon}({\left\{\left(u,s_{u}\right)\right\}}_{u\in V},t)\to s$, as |V|≥t, for all users in *V*, they have at least *t* points on the polynomial $p_{s}\left(x\right)$; through Lagrange interpolation, they can reconstruct the polynomial and output $s=p_{s}\left(0\right)$.

The correctness of the scheme requires that, for any s∈F, any t,n with 1≤t≤n, and any U⊆F, where |U|=n, if ${\left\{\left(u,s_{u}\right)\right\}}_{u\in U}$ is the output of algorithm SS.share(s,t,U), V⊆U and |V|≥t, then $\mathrm{SS}.\mathrm{recon}({\left\{\left(u,s_{u}\right)\right\}}_{u\in V},t)=s$ holds.

The security (against a semi-honest adversary) of the scheme requires that, for any $s,s^{\prime }\in F$, and any V⊆U, such that |V|<t, we have $\left\{{\left\{\left(u,s_{u}\right)\right\}}_{u\in U}\leftarrow \mathrm{SS}.\mathrm{share}\left(s,t,U\right):{\left\{\left(u,s_{u}\right)\right\}}_{u\in V}\right\}\equiv \left\{{\left\{\left(u,s_{u}\right)\right\}}_{u\in U}\leftarrow \mathrm{SS}.\mathrm{share}\left(s^{\prime },t,U\right):{\left\{\left(u,s_{u}\right)\right\}}_{u\in V}\right\}$ holding, where ≡ represents the fact that the two distributions are identical. In other words, any less than *t* shares of a secret, *s*, contain no information about *s*, as the distribution of these shares is indistinguishable from the distribution of the corresponding shares of any element $s^{\prime }\in F$.

Shamir’s *t*-out-of-*n* secret sharing can be proven to be correct and semi-honestly secure. We specify that, when a user wants to share their secret with other users, this user plays the role of the dealer in a secret sharing scheme; the secret sharing polynomial (coefficients) is chosen by this user.

### 2.2. Key Agreement

A key agreement scheme consists of three algorithms, which are the parameterization algorithm, the generating algorithm and the agreement algorithm. The parameterization algorithm KA.param(λ)→pp takes security parameter λ as input and outputs some public parameters, pp, for the scheme. The generating algorithm $\mathrm{KA}.\mathrm{gen}\left(pp\right)\to \left(s_{u}^{sk},s_{u}^{pk}\right)$ takes as input the public parameters pp and allows any user, *u*, to generate a private–public key pair. The agreement algorithm $\mathrm{KA}.\mathrm{agree}\left(s_{u}^{sk},s_{v}^{pk}\right)\to s_{u,v}$ allows user *u* to combine his private key $s_{u}^{sk}$ with the other user *v*’s private key $s_{v}^{pk}$ (generated by the same public parameter pp) to obtain a private shared key $s_{u,v}$ between *u* and *v*.

More specifically, we will use the Diffie–Hellman key agreement scheme [23]. The public parameter that the parameterization algorithm outputs is pp=(G,p,g,H), where G is a cyclic group with prime order *p* and a generator, *g*, and *H* is a hash function. The generating algorithm $\mathrm{KA}.\mathrm{gen}\left(G,p,g,H\right)\to \left(a_{u},g^{a_{u}}\right)$ samples a random element, $a_{u}$, in $Z_{p}$ as the private key of user *u*, and the corresponding public key is $g^{a_{u}}$. The agreement algorithm is then as $\mathrm{KA}.\mathrm{agree}\left(a_{u},g^{a_{v}}\right)\to s_{u,v}=H\left({\left(g^{a_{v}}\right)}^{a_{u}}\right)$.

The correctness of the scheme requires that, for any users *u* and *v*, and for any key pairs generated by the same public parameter, we have $\mathrm{KA}.\mathrm{agree}\left(s_{u}^{sk},s_{v}^{pk}\right)=\mathrm{KA}.\mathrm{agree}\left(s_{v}^{sk},s_{u}^{pk}\right)$. That is, $s_{u,v}=s_{v,u}$ is the private shared key between *u* and *v*.

The security of the scheme is based on the hardness of decisional Diffie–Hellman(DDH) problem.

**Definition** **1.** *(G,p,g) is a triple, and G is a cyclic group of order p with generator g. We say the DDH problem is hard relative to (G,p,g) if, for all probabilistic, polynomial-time algorithms A, there exists a negligible function, negl, such that*

$$|\mathrm{Pr}\left[A\left(G,p,g,g^{x},g^{y},g^{z}\right)=1\right]-\mathrm{Pr}\left[A\left(G,p,g,g^{x},g^{y},g^{xy}\right)=1\right]|\;\leq negl\left(\lambda \right)$$

*where x,y,z are random elements in $Z_{p}$.*

That is to say that, in the case in which $g^{x},g^{y}\in G$ are known, but $x,y\in Z_{p}$ is kept unknown, it is impossible to distinguish $g^{xy}$ and a random element in G. For the triple of the prime order cyclic group, the DDH problem can be proven to be hard.

**Definition** **2.** Consider a probabilistic experiment below, where M is a probabilistic polynomial-time algorithm, b is a randomly chosen bit, KA.param(λ)→(G,p,g,H) is a parameterization algorithm, $H:{\left\{0,1\right\}}^{*}\to {\left\{0,1\right\}}^{\lambda }$ is a hash function, and λ is the security parameter.

*Diffie–Hellman key agreement experiment ${\mathrm{DH}-\mathrm{EXP}}_{G,M}\left(\lambda \right)$:*

*1. (G,p,g,H)←KA.param(λ).*

*2. Randomly choose $x,y\in Z_{p}$*

*3. If b=0, then let $s=H(g^{xy})$; if b=1, then take s as a random element in ${\left\{0,1\right\}}^{\lambda }$.*

*4. $M\left(G,p,g,H,g^{x},g^{y},s\right)\to b^{\prime }$.*

*5. If $b=b^{\prime }$, outputs 1; if $b\neq b^{\prime }$, outputs 0.*

*The Diffie–Hellman key exchange scheme is secure in the presence of semi-honest adversaries if, for any probabilistic polynomial-time algorithm, M, there exists a negligible function, negl, such that*

$$|\mathrm{Pr}\left[{\mathrm{DH-EXP}}_{G,M}\left(\lambda \right)=1\right]-\frac{1}{2}|\;\leq negl\left(\lambda \right)$$

In other words, in the case in which the public keys $g^{a_{u}}$ and $g^{a_{v}}$ of users *u* and *v* are known, but their private keys $a_{u}$ and $a_{v}$ are kept unknown, the shared key $s_{u,v}=s_{v,u}$ that they have agreed on is indistinguishable from a uniformly distributed random string.

**Theorem** **1.** *When the DDH problem is hard relative to the output triple (G,p,g) of the algorithm KA.param(λ), the Diffie–Hellman key agreement scheme is secure in the presence of semi-honest adversaries.*

### 2.3. Bilinear Map

Bilinear maps based on Weil pairing [27] are used as cryptographic primitives. A bilinear map can be represented as a map, $e:G\times G\to G_{T}$, where both G and $G_{T}$ are multiplicative cyclic groups with the same prime order *p*. The generator of group G is *g*. Bilinear map *e* has the following properties:

(1) Computability: for any $g_{1},g_{2}\in G$, $e(g_{1},g_{2})$ can be computed efficiently.

(2) Bilinearity: for any $a,b\in Z_{p}^{*}$, and any $g_{1},g_{2}\in G$, we have

$$e\left(g_{1}^{a},g_{2}^{b}\right)=e{\left(g_{1}^{a},g_{2}\right)}^{b}=e{\left(g_{1},g_{2}^{b}\right)}^{a}=e{\left(g_{1},g_{2}\right)}^{ab}$$

(3) Non-degeneracy: for the generator *g* of group G, e(g,g)≠1.

## 3. Model Statement

**Figure 1:**
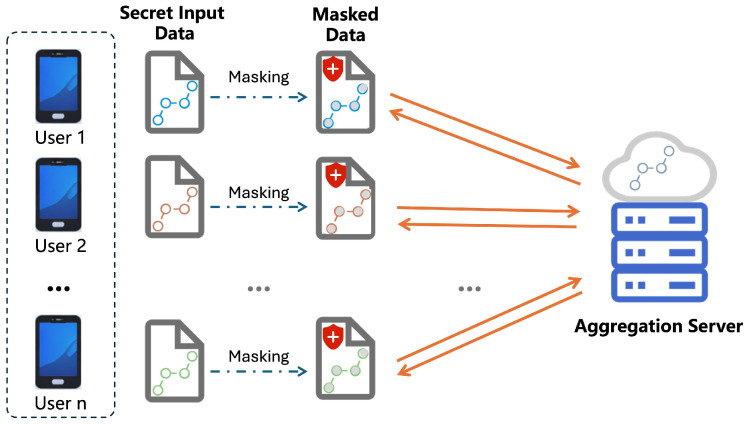
Communication Model of Secure Aggregation.

The model of the secure aggregation problem that we want to discuss consists of a trusted authority (TA), a central server (*S*) and a large number of users. The communication model of secure aggregation is shown in Figure 1. The trusted authority only produces and broadcasts the public parameter of the protocol but will not participate in the subsequent interactions between server and users. Every user has a private authenticated communication channel with the server, while users are not able to communicate directly. Each user has a high-dimensional private vector that belongs to $Z_{R}^{m}$. Users are allowed to drop out at any time of the protocol execution, but once a user has dropped out, they cannot go back to the protocol again. The server can always see who has dropped out. The dropout set is assumed to be exogenous and cannot be adversarially influenced. Whenever the number of online users is less than the secret sharing threshold parameter *t*, the protocol will abort with no output.

Correctness requires that the final output of the server is the additive/multiplicative aggregation result (sum/component-wise product) of some users’ private vector, as these users have not dropped out before they send the message related to private vector to the server. On account of correctness parameter η, we only need to guarantee that the output equals the correct product with probability $\geq 1-\frac{1}{2^{\eta }}$.

For security, we only consider the case against semi-honest adversaries. Semi-honest adversaries are among the participants in the protocol, who strictly follow the protocol, and meanwhile observe what they have sent and received in the execution, and interflow with each other, hoping to get information about honest users’ private vector. We will give a general definition of the security of the protocol against semi-honest adversaries in the following. Loosely speaking, the definition states that a protocol is Δ-private if the view of up to Δ corrupted parties in a real protocol execution can be generated by a simulator given only the corrupted parties’ inputs and outputs.

For a general *n*-ary function, $f:{\left({\left\{0,1\right\}}^{*}\right)}^{n}\to {\left({\left\{0,1\right\}}^{*}\right)}^{n}$, to be securely computed by *n* parties $P_{1},\ldots ,P_{n}$, assume that π is a protocol that achieves secure computation of *f*. During an execution of the protocol π on inputs $\vec{x}=\left(x_{1},\ldots ,x_{n}\right)$, the view of the *i*th party $P_{i}$, denoted as ${\mathrm{VIEW}}_{i}^{\pi }\left(\vec{x}\right)$, is defined to be $\left(x_{i},r_{i};m_{i,1},\ldots ,m_{i,k}\right)$, where $x_{i}$ is $P_{i}$’s private input, $r_{i}$ is the internal coin tosses generated by $P_{i}$, and $m_{i,j}$ is the *j*th message that was received by $P_{i}$ in the protocol execution. For every $I=\left\{i_{1},\ldots ,i_{l}\right\}\subset \left\{1,\ldots ,n\right\}$, denote ${\mathrm{VIEW}}_{I}^{\pi }\left(\vec{x}\right)=\left({\mathrm{VIEW}}_{i_{1}}^{\pi }\left(\vec{x}\right),\ldots ,{\mathrm{VIEW}}_{i_{l}}^{\pi }\left(\vec{x}\right)\right)$. The output of all parties from an execution of π on inputs $\vec{x}$ is denoted as ${\mathrm{OUTPUT}}^{\pi }\left(\vec{x}\right)$; observe that the output of each party can be computed from its own (private) view of the execution.

**Definition** **3.** *Let* $f:{\left({\left\{0,1\right\}}^{*}\right)}^{n}\to {\left({\left\{0,1\right\}}^{*}\right)}^{n}$ *be a deterministic n-ary functionality, and let π be a protocol. We say that π is* Δ*-private for computing f if, for every* $\vec{x}\in {\left({\left\{0,1\right\}}^{*}\right)}^{n}$*, we have*

$${\mathrm{OUTPUT}}^{\pi }\left(x_{1},\ldots ,x_{n}\right)=f\left(x_{1},\ldots ,x_{n}\right)$$

*and there exists a probabilistic polynomial-time algorithm,* SIM*, such that, for every* I⊂[n] *with* |I|≤Δ *and every* $\vec{x}\in {\left({\left\{0,1\right\}}^{*}\right)}^{n}$*, it holds that*

$$\left\{\mathrm{SIM}\left(I,{\vec{x}}_{I},f_{I}\left(\vec{x}\right)\right)\right\}\equiv_{c}\left\{{\mathrm{VIEW}}_{I}^{\pi }\left(\vec{x}\right)\right\}$$

*where* $\equiv_{c}$ *represents the fact that two random variables are computationally indistinguishable.*

In other words, for any set, *I*, of corrupt parties (adversaries), except their inputs and outputs, they could not obtain any information from what they have sent and received in the protocol execution; in particular, they have no information about honest users’ private vector.

For secure additive/multiplicative aggregation, the *n*-ary function *f* is restricted to a special form. The server and users are the participants of the protocol, users only input their private vector but have no outputs, and the server has no input but outputs the sum/component-wise product of online users’ private vector. Our protocols achieve *t*-privacy, where *t* is the threshold parameter of secret sharing.

## 4. Secure Multiplicative Aggregation

In this section, we will first present the high-level idea of our secure multiplicative aggregation protocol and then give a full description of the protocol and prove its correctness and security. Finally, we will analyze the theoretical overhead of our secure multiplicative aggregation protocol.

### 4.1. Intuition

**Figure 2:**
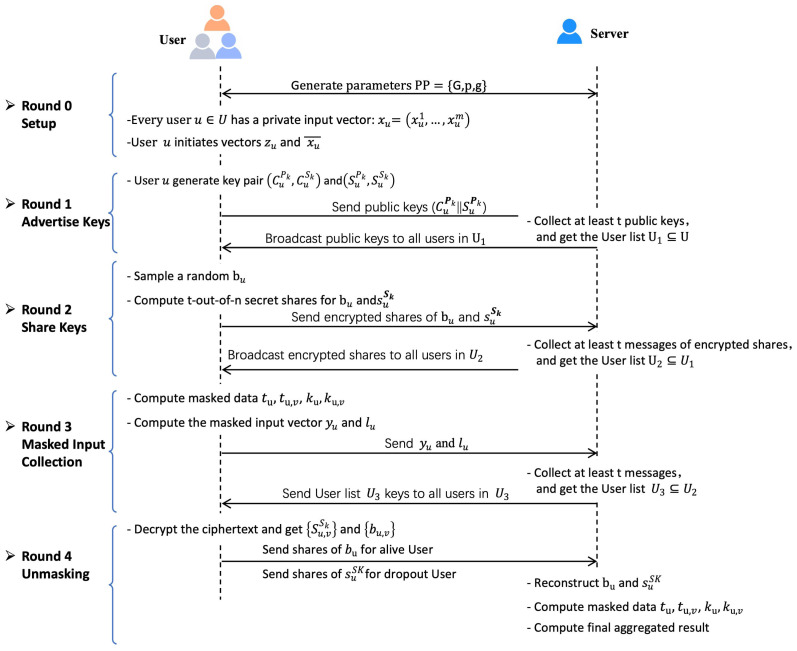
Framework of Multiplicative Aggregation.

We provide the framework of the secure multiplicative aggregation protocol in Figure 2. A promising idea that realizes secure multiplicative aggregation using the secure additive aggregation protocol is to process each component of each input vector with a logarithm to obtain the inputs of secure aggregation and compute the exponentiation of the output of secure aggregation to get the final output. However, as the space of users’ private input vectors is $Z_{R}^{m}$, the components of input vectors may be 0 components, leading to failures when taking the logarithm. Therefore, we mainly focus on dealing with possible 0 components in input vectors of secure multiplicative aggregation.

One important fact is that, for a fixed component with index j∈[m] in *m*-dimension vectors, if there exists one of *n* private input vectors from *n* users, such that its component with index *j* is equal to 0, then the *j*th component of final output must be 0; conversely, when *R* is a prime number, if all *n* private input vectors from *n* users have their components with index *j* to be nonzero, then the *j*th component of final output must be nonzero. This means that the indices of 0 components in input vectors fully determine the indices of 0 components in the output result. We leverage the property to design a secure multiplicative aggregation protocol. On the one hand, each user is allowed to obtain a new input vector by replacing 0 components in its input vectors with uniformly random non-zero elements from $Z_{R}^{*}$. Secure multiplicative aggregation with new input vectors as inputs can be realized using the idea of taking a logarithm, as well as the secure aggregation protocol, since new input vectors have no 0 components. On the other hand, users have to provide information about the indices of 0 components in their input vectors to the server, which can be used to identify the indices of 0 components in the server’s final output.

To keep the indices of 0 components in users’ inputs unknown to the server (otherwise, the server learns extra information about honest users’ private input), while letting the server learn the indices of 0 components in the component-wise product result, we exploit the secure aggregation protocol with auxiliary vectors as inputs. Specifically, each user initializes an auxiliary vector that belong to $Z_{q}^{m}$, with all components being 0. For each index of 0 component in the user’s input, the component in the auxiliary vector with the same index is replaced by a non-zero uniformly random element from $Z_{q}$. In this way, we store the information about the index of the 0 component in the initial input into the auxiliary vector. The server obtains the output of the secure aggregation of auxiliary vectors that belongs to $Z_{q}^{m}$. For a fixed component with index j∈[m] in *m*-dimension vectors, if the *j*th component in the secure aggregation result is non-zero, then the *j*th component of the secure multiplicative aggregation output must be 0; conversely, if the secure aggregation result has a 0 component with index *j*, then the *j*th component of the secure multiplicative aggregation output is non-zero with high probability. Finally, the output is obtained with 0 components from the secure aggregation result of auxiliary vectors and non-zero components from the secure multiplicative aggregation of new input vectors that replace 0 components with uniformly random non-zero elements from $Z_{R}^{*}$. According to the security of secure additive aggregation, the server learns nothing about honest users’ auxiliary vectors; thus, the indices of 0 components in honest users’ inputs are kept private to the server.

**Figure 3:**
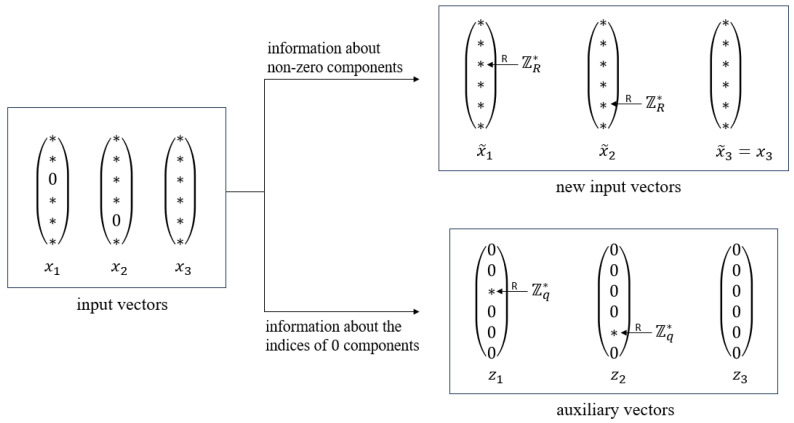
Our main intuition for dealing with 0 components in input vectors (* means the element is non-zero).

Here, we give a toy example in Figure 3 to help explain our main intuition more clearly (for simplicity, we assume that there is no dropout). Consider three input vectors, $x_{1},x_{2},x_{3}$, of dimension 6, where 0 only appears in the third component of $x_{1}$ and the fifth component of $x_{2}$. On the one hand, the appearance of 0 results in the failure of taking the logarithm, which directly turns secure multiplicative aggregation into secure additive aggregation; on the other hand, since *R* is a prime number, it is clear that 0 will appear in the third and the fifth components of the final result. Therefore, we construct new input vectors, ${\tilde{x}}_{1},{\tilde{x}}_{2},{\tilde{x}}_{3}$, from $x_{1},x_{2},x_{3}$ in such a way that each 0 component is replaced by a uniform random element from $Z_{R}^{*}$, and each non-zero component remains the same to store the information about non-zero components; and we construct auxiliary vectors, $z_{1},z_{2},z_{3}$, in such a way that each 0 component is replaced by a uniform random element from $Z_{q}^{*}$, and each non-zero component is replaced by 0 to store the information about the indices of 0 components in input vectors. Now that we execute secure multiplicative aggregation to ${\tilde{x}}_{1},{\tilde{x}}_{2},{\tilde{x}}_{3}$ (since there is no 0 component in ${\tilde{x}}_{1},{\tilde{x}}_{2},{\tilde{x}}_{3}$, it can be reduced to secure additive aggregation by taking the logarithm) and secure additive aggregation to $z_{1},z_{2},z_{3}$ simultaneously. The indices of non-zero components $z_{1}+z_{2}+z_{3}$ shows the indices of 0 in the result of $x_{1}\cdot x_{2}\cdot x_{3}$, which is 3 and 5 in the example. For other non-zero components, the result of $x_{1}\cdot x_{2}\cdot x_{3}$ is exactly the same as that of ${\tilde{x}}_{1}\cdot {\tilde{x}}_{2}\cdot {\tilde{x}}_{3}$. The security of secure aggregation guarantees that the server learns nothing about ${\tilde{x}}_{1},{\tilde{x}}_{2},{\tilde{x}}_{3}$ and $z_{1},z_{2},z_{3}$ except ${\tilde{x}}_{1}\cdot {\tilde{x}}_{2}\cdot {\tilde{x}}_{3}$ and $z_{1}+z_{2}+z_{3}$; thus, the non-zero components and the indices of the 0 component in input vectors are hidden from the server.

### 4.2. Secure Multiplicative Aggregation Protocol

Before giving the construction, we provide in Table 1 the parameters we will use throughout the protocols. We have to point out that, in our constructions, the threshold parameter of the number of online users and the threshold parameter of the number of parties corrupted by a semi-honest adversary (the parties can be users or the server) are required to be the same as the secret sharing threshold parameter *t*, since we use secret sharing as a building block.

A complete description of the secure multiplicative aggregation protocol is as follows.

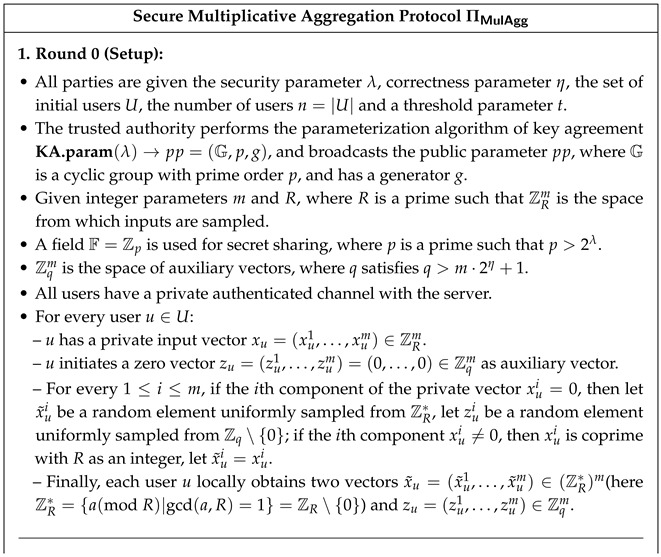

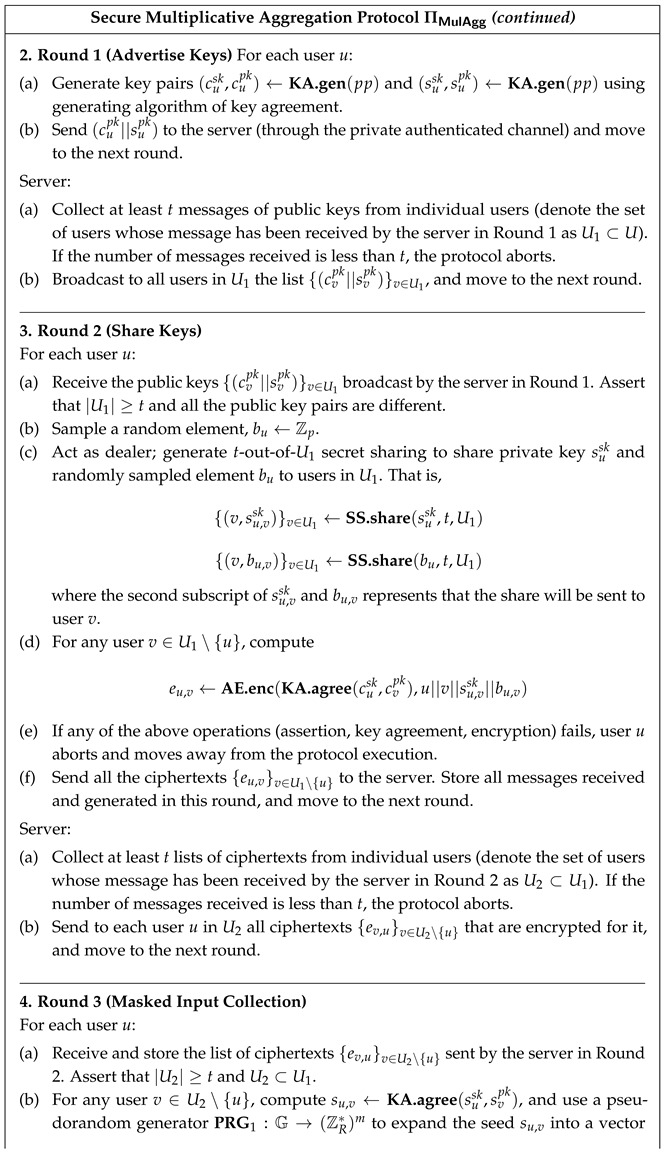

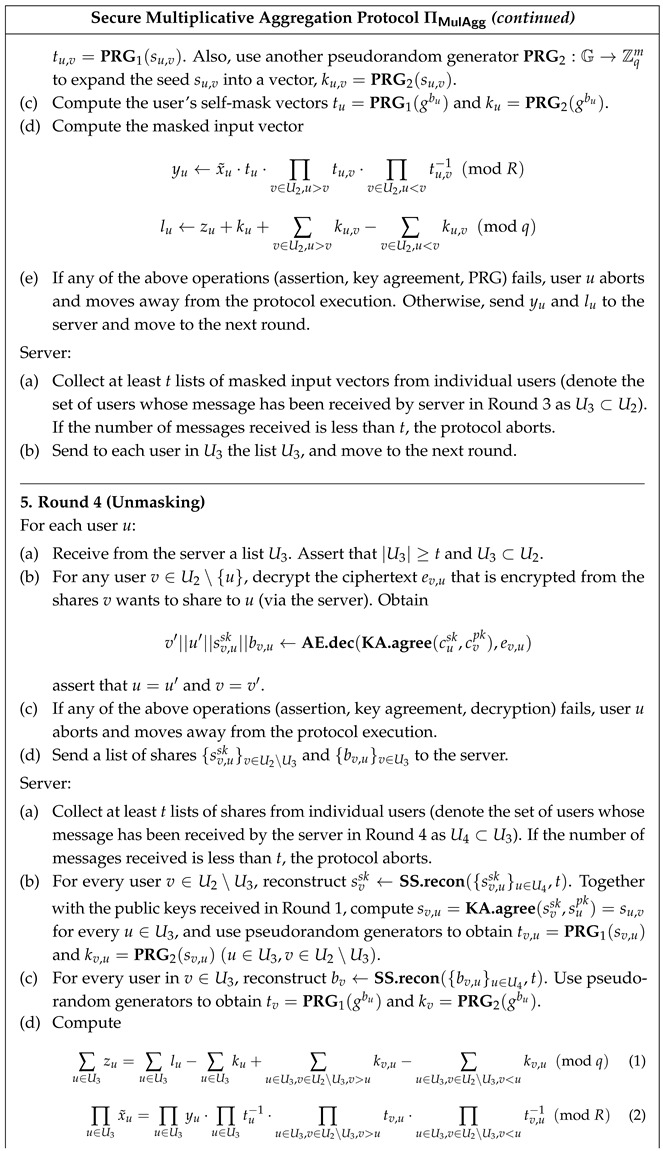

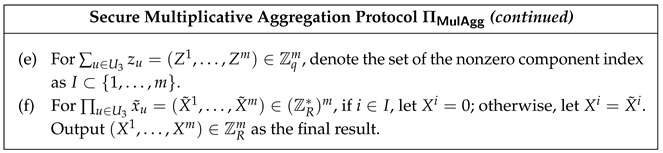

**Theorem** **2** (Correctness of the protocol $\Pi_{\mathrm{MulAgg}}$)**.** *If the protocol $\Pi_{\mathrm{MulAgg}}$ has an output, then the output will equal the correct product of private vectors with probability $\geq 1-\frac{1}{2^{\eta }}$.*

The proof of the theorem is given in Appendix C.

Next, we talk about the security of the protocol $\Pi_{\mathrm{MulAgg}}$. We will prove that the protocol is secure against semi-honest adversaries, no matter when the users drop out during the protocol execution. In other words, for the protocol $\Pi_{\mathrm{MulAgg}}$ with threshold parameter *t*, the joint view of at most *t* participants in the protocol (participants including the server and all users) does not leak any information about the other users’ private inputs, where these parties are called semi-honest parties.

In the secure multiplicative aggregation protocol, there is a central server, S, a set of initial users *U* with |U|=n, and a threshold parameter, *t*. Users are allowed to drop out at any time during the protocol execution; denote the set of users whose message has been received by the server in Round *i* as $U_{i}$. We have $U⊇U_{1}⊇U_{2}⊇U_{3}⊇U_{4}$. Each user, *u*, has a private input vector, $x_{u}$; denote $x_{U^{\prime }}={\left\{x_{u}\right\}}_{u\in U^{\prime }}$, where $U^{\prime }$ can be any subset of users. The same as the definition in the model statement, ${\mathrm{VIEW}}_{u}\left(x_{U}\right)$ represents the view of party u∈U∪{S} when the protocol runs with inputs $x_{U}$. Define C⊂U∪{S} as the set of semi-honest parties, and then ${\mathrm{VIEW}}_{C}\left(x_{U},U_{1},U_{2},U_{3},U_{4}\right)$ is the joint view of semi-honest adversaries during the protocol execution.

Firstly, we will consider the case where semi-honest parties are a set of users, that is, C⊂U.

**Theorem** **3** (Security of the protocol $\Pi_{\mathrm{MulAgg}}$ against semi-honest users)**.** *There exists a probabilistic polynomial-time simulator, SIM, such that for any security parameter λ, threshold parameter t, set of users U with |U|≥t, input vectors $x_{U}$, sets of online users $U⊇U_{1}⊇U_{2}⊇U_{3}⊇U_{4}$ and set of semi-honest parties C⊆U with |C|≤t, the output of SIM is perfectly indistinguishable from ${\mathrm{VIEW}}_{C}$ as random variables; that is,*

$${\mathrm{VIEW}}_{C}\left(x_{U},U_{1},U_{2},U_{3},U_{4}\right)\equiv {\mathrm{SIM}}_{C}\left(x_{C},U_{1},U_{2},U_{3},U_{4}\right)$$

**Proof.**  For any user u∈C, all they have received during the protocol execution are public keys ${\left\{c_{v}^{pk},s_{v}^{pk}\right\}}_{v\in U_{1}\setminus \left\{u\right\}}$, ciphertexts ${\left\{e_{v,u}\right\}}_{v\in U_{2}\setminus \left\{u\right\}}$ and a list of $U_{3}$. Note that these messages do not depend on the inputs of the honest users. In fact, the only values sent by each honest user *v* that depend on their private input are $y_{v}$ and $l_{v}$, which they sent to the server in Round 3, but the server is honest. So, when knowing the inputs $x_{C}$ of user set C, the simulator SIM can be defined to use zero vectors for the inputs of all honest users and to use $x_{u}\left(u\in C\right)$ for the inputs of semi-honest users *u* and then to simulate the execution of the secure multiplicative aggregation protocol. The simulator outputs the joint view of users in C in the simulation process, which is perfectly indistinguishable from ${\mathrm{VIEW}}_{C}$. □

When the server is semi-honest, it can share what it has received in the protocol execution with other semi-honest users. We show that the view of any such group of semi-honest parties can be simulated by a simulator, which is given the inputs of the users in that group and the final output of the server.

**Theorem** **4** (Security of the protocol $\Pi_{\mathrm{MulAgg}}$ against semi-honest server and users)**.** *There exists a probability polynomial-time simulator, SIM, such that, for any security parameter λ, threshold parameter t, set of users U with |U|≥t, input vectors $x_{U}$, sets of online users $U⊇U_{1}⊇U_{2}⊇U_{3}⊇U_{4}$ and set of semi-honest parties C⊆U∪{S} with |C∖{S}|<t, the output of SIM is perfectly indistinguishable from ${\mathrm{VIEW}}_{C}$ as random variables; that is,*

$${\mathrm{VIEW}}_{C}\left(x_{U},U_{1},U_{2},U_{3},U_{4}\right)\equiv {\mathrm{SIM}}_{C}\left(x_{C},X,U_{1},U_{2},U_{3},U_{4}\right)$$

*where*

$$X=\left\{\begin{array}{cc} \left(X^{1},\ldots ,X^{m}\right) & \mathrm{if}\;\left|U_{3}\right|\geq \;t\\ ⊥ & \mathrm{otherwise} \end{array}\right.$$

The proof of the Theorem is based on the security of the Diffie–Hellman key agreement, the IND-CPA security of the authenticated encryption scheme, the security of secret sharing and the security of the pseudorandom generator. It is given in Appendix D.

### 4.3. Analysis of Theoretical Overhead

The theoretical overhead of our protocol will be analyzed in terms of computational, communication, and storage costs.

– The computation cost for a *user*, *u*, of our $\Pi_{\mathrm{MulAgg}}$ protocol contains: (i) computing two vectors $z_{u}$ and ${\tilde{x}}_{u}$ associated with a private input vector, (ii) performing 2(n−1) key agreements with other users, (iii) creating *t*-out-of-*n* secret sharing of $s_{u}^{sk}$ and $b_{u}$, (iv) generating $t_{u},t_{u,v}$ and $k_{u},k_{u,v}$ using ${\mathrm{PRG}}_{1}$ and ${\mathrm{PRG}}_{2}$, and also computing $y_{u}$ and $l_{u}$. Each user’s total computation cost is $O(n^{2}+nm\log m)$.
– The communication cost for a *user u* contains: (i) sending their public keys, $s_{u}^{pk},c_{u}^{pk}$, and receiving (from the server) other users’ public keys, (ii) sending encrypted secret shares, $e_{u,v}$, and receiving $e_{v,u}$ from the server, (iii) sending masked input vectors, $y_{u}$ and $l_{u}$, to the server, and (iv) sending the server decrypted secret shares $s_{v,u}^{sk}$ and $b_{v,u}$. Each user’s total communication cost is O(n+mlogm).
– The storage cost for a *user u* contains: (i) storing private input vector $x_{u}$ and two vectors $z_{u},{\tilde{x}}_{u}$ derived from it and (ii) storing all users’ public keys, their own private keys and encrypted secret shares $e_{v,u}$. Each user’s total storage cost is O(n+mlogm).
– The computation cost for the *server* of our $\Pi_{\mathrm{MulAgg}}$ protocol contains: (i) reconstructing *t*-out-of-*n* secrets (one for each user) using Lagrange interpolation, (ii) computing the masks $t_{u},t_{u,v}$ and $k_{u},k_{u,v}$, obtaining $\prod_{u\in U_{3}}{\tilde{x}}_{u}$ and $\sum_{u\in U_{3}}z_{u}$, and finally outputting $\left(X^{1},\ldots ,X^{m}\right)$. The server’s total computation cost is $O(n^{2}m\log m)$. Note that, in general, the reconstructions of O(n) secrets by Lagrange interpolation require $O(n^{3})$ computation, as, for any secret reconstruction $\mathrm{SS}.\mathrm{recon}({\left\{\left(u,s_{u}\right)\right\}}_{u\in U^{\prime }},t)\to s$, the server needs to compute
  $$s=L\left(0\right)=\sum_{u\in U^{\prime }}s_{u}\prod_{v\in U^{\prime }\setminus \left\{u\right\}}\frac{v}{v-u}\;\;\left(\mathrm{mod}\;q\right)$$
  which costs $O(n^{2})$ computation. Actually, in our $\Pi_{\mathrm{MulAgg}}$ protocol, where every user has identity fixed at the very beginning, the total time to reconstruct all secrets can be reduced, as the set $U^{\prime }$ is always $U_{4}$. The server only needs to precompute the Lagrange basis polynomials (values at 0 of the polynomials)
  $$L_{u}=\prod_{v\in U_{4}\setminus \left\{u\right\}}\frac{v}{v-u}\;\;\left(\mathrm{mod}\;q\right)$$
  which costs $O(n^{2})$ in computation, and then perform linear computation $L\left(0\right)=\sum_{u\in U_{4}}s_{u}L_{u}\;\;\left(\mathrm{mod}\;q\right)$ to reconstruct secrets. In this way, O(n) reconstructions take $O(n^{2})$ time.
– The communication cost for the *server* contains: (i) sending and receiving messages between users as the mediation, (ii) receiving the masked input vectors $y_{u}$ and $l_{u}$ sent by user *u*, and also the decrypted secret shares. The server’s total communication cost is $O(n^{2}+nm\log m)$.
– The storage cost for the *server* contains: (i) storing all users’ public keys, (ii) storing decrypted secret shares sent by users, and (iii) storing masked input vectors $y_{u}$ and $l_{u}$, so as to do addition or multiplication. The server’s total storage cost is $O(n^{2}+m\log m)$.

## 5. Key Reusable Secure Aggregation

Consider the case in which the set *U* of initial users is fixed, each user has some private inputs, and they are willing to perform secure additive/multiplicative aggregation many times on different inputs. Such a multi-round setting can be adapted to federated learning [1], where multiple rounds of training are needed until the model converges.

It is clear that using a state-of-the-art secure additive aggregation protocol [24] or the secure multiplicative aggregation protocol $\Pi_{\mathrm{MulAgg}}$ multiple times can solve the problem naively. For both of the protocols, to guarantee the security, all users u∈U have to generate a new public–private key pair, $\left(s_{u}^{sk},s_{u}^{pk}\right)$, again in Round 1 every time they perform the secure additive/multiplicative aggregation execution. This is because, if $\left(s_{u}^{sk},s_{u}^{pk}\right)$ is reused, it is possible that *u* is in the set $U_{2}\setminus U_{3}$ defined by the additive/multiplicative aggregation execution last time, and so, according to the protocol, the server has reconstructed this private key $s_{u}^{sk}$ in Round 4; therefore, the server has all the pairwise masks of *u*. It is also possible that *u* is in the set $U_{3}$ defined by the additive/multiplicative aggregation execution this time; so, according to the protocol, the server is able to know the self masks of *u*. With pairwise masks and self masks of *u*, the server can obtain the private input of user *u* from the masked input, which breaks the security.

Therefore, what we accept is to construct additive/multiplicative aggregation protocols that allow users to *reuse* their public–private key pairs generated in Round 1. In this section, we will present a key reusable secure additive aggregation protocol based on the construction of Bonawitz et al. [24] and a key reusable multiplicative aggregation protocol based on the construction of $\Pi_{\mathrm{MulAgg}}$ in Section 4.

### 5.1. Key Reusable Secure Additive Aggregation

As mentioned above, the reason why the public–private key pairs $\left(s_{u}^{sk},s_{u}^{pk}\right)$ generated in Round 1 of the protocol of Bonawitz et al. [24] cannot be reused is that the server will reconstruct the private key $s_{u}^{sk}$ for $u\in U_{2}\setminus U_{3}$ in Round 4, which is then used to compute the pairwise masks to be removed from $\sum_{u\in U_{3}}y_{u}$.

**Figure 4:**
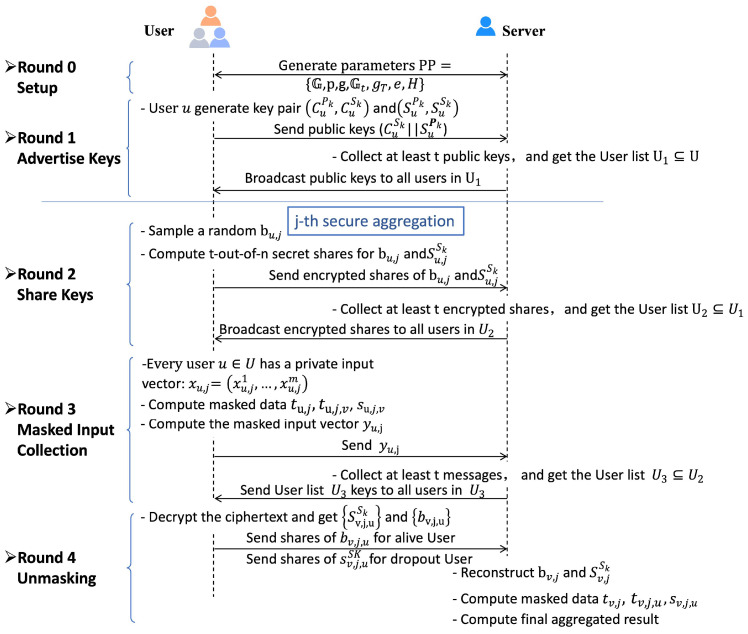
Framework of Key Reusable Secure Additive Aggregation.

To maintain the privacy of the long-term private key $s_{u}^{sk}$, we construct a one-time private key, $s_{u,j}^{sk}$, for *j*th additive aggregation execution using $s_{u}^{sk}$ and the index *j* as $s_{u,j}^{sk}=H{\left(j\right)}^{s_{u}^{sk}}$. The one-time private key $s_{u,j}^{sk}$ is shared among users, and the pairwise masks are now computed using a bilinear map, rather than key agreement, to avoid the use of a one-time public key. The bilinearity of the bilinear map ensures that both users *u* and *v* can compute the pairwise mask $e\left(s_{u,j}^{sk},s_{v}^{pk}\right)=e\left(s_{v,j}^{sk},s_{u}^{pk}\right)$ using their one-time private keys and the long-term public keys. For user $u\in U_{2}\setminus U_{3}$ in *j*th execution, the server will reconstruct their one-time private key $s_{u,j}^{sk}$ in Round 4 to obtain the pairwise masks to be removed. Under the DDH assumption, the server with one-time private key $s_{u,j}^{sk}$ learns nothing about the long-term private key $s_{u}^{sk}$; thus, $s_{u}^{sk}$ can be reused. We provide the framework of our key reusable secure additive aggregation protocol in Figure 4.

A complete description of the key reusable secure additive aggregation protocol is as follows.

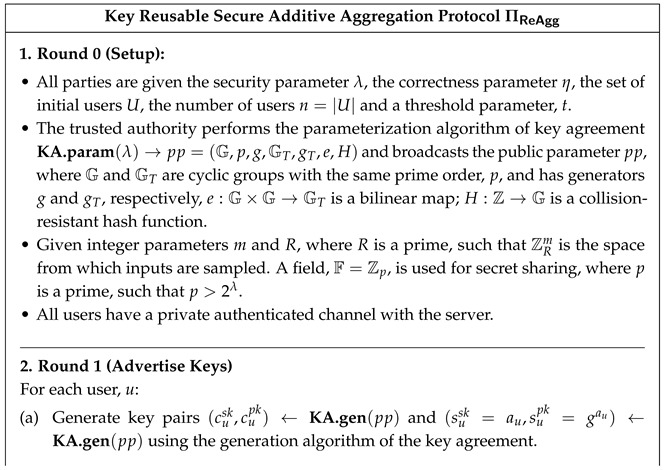

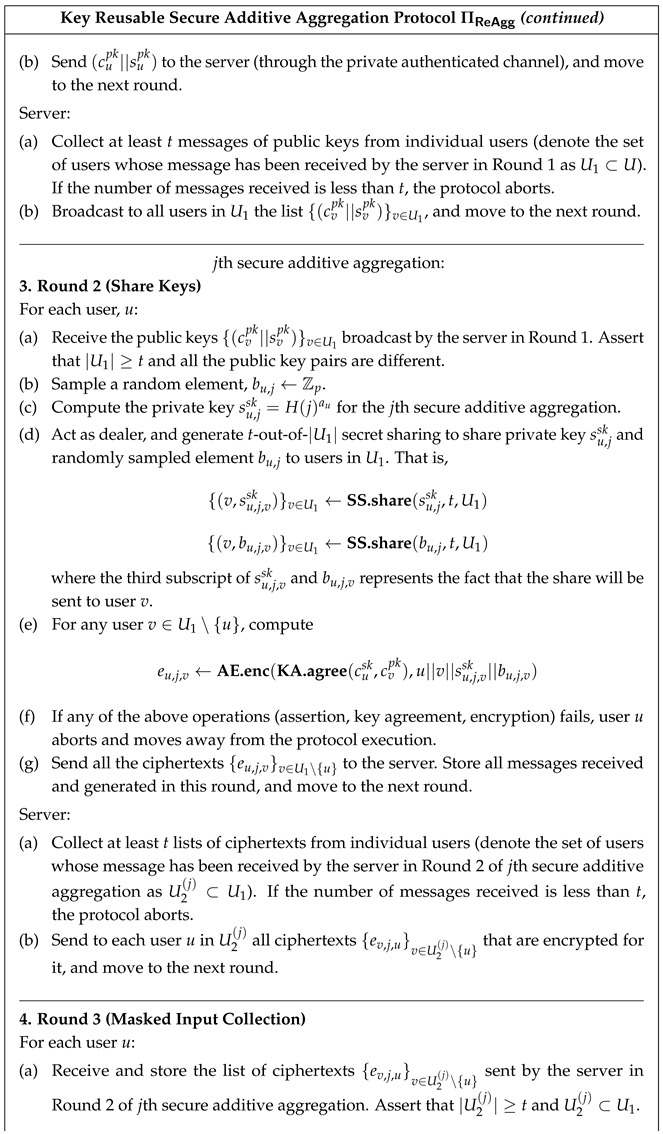

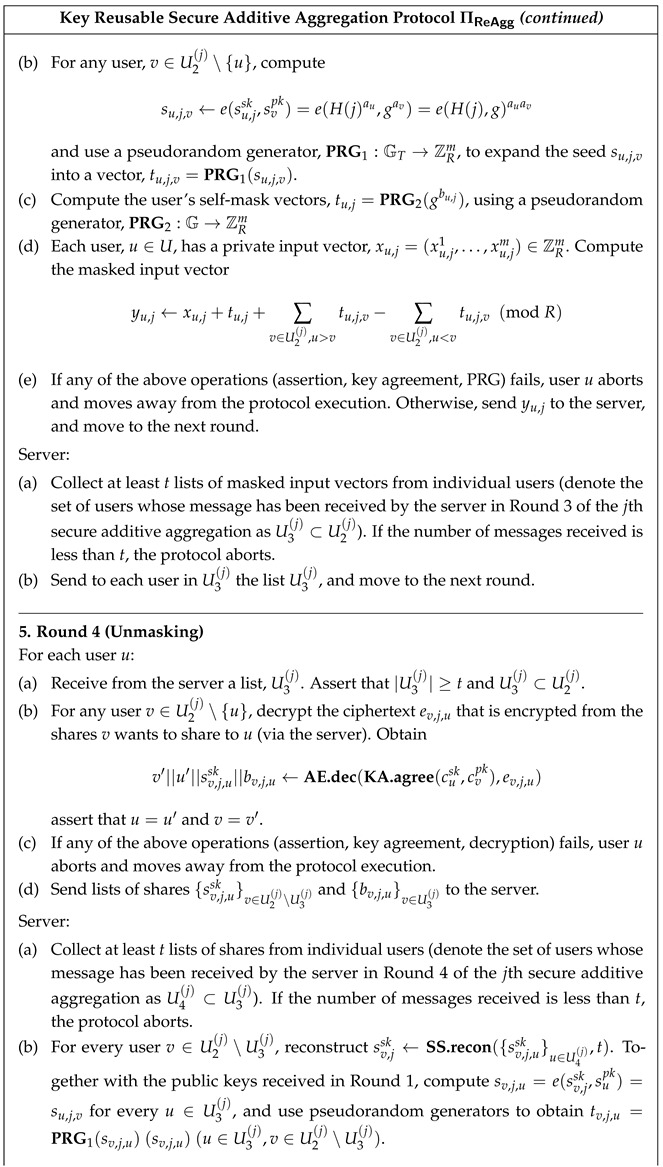

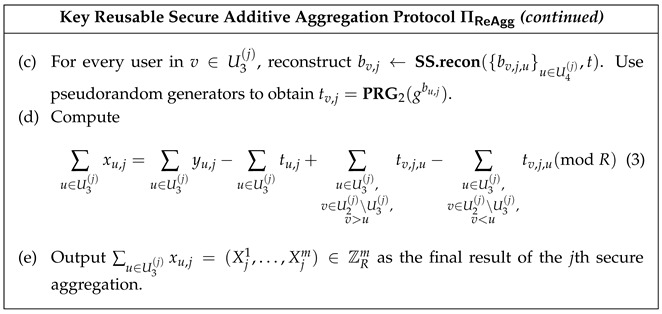

**Theorem** **5** (Correctness of the protocol $\Pi_{\mathrm{ReAgg}}$)**.** *In the protocol $\Pi_{\mathrm{ReAgg}}$, for each additive aggregation execution, if it has an output, then the output will be equal to the correct sum of private input vectors.*

The proof of the Theorem is given in Appendix E.

Next, we talk about the security of the protocol $\Pi_{\mathrm{ReAgg}}$. We will prove that the protocol is secure against static semi-honest adversaries, no matter when the users drop out during the protocol execution.

In the key reusable secure additive aggregation protocol, there is a central server, S, a set of initial users, *U*, with |U|=n, and a threshold parameter, *t*. Users are allowed to drop out at any time of the entire protocol and rejoin the protocol for a new additive aggregation execution; denote the set of users whose message has been received by the server in Round *i* of *j*th secure additive aggregation as $U_{i}^{\left(j\right)}$. We have $U⊇U_{1}⊇U_{2}^{\left(j\right)}⊇U_{3}^{\left(j\right)}⊇U_{4}^{\left(j\right)}$. Denote $U_{r}\left(r=2,3,4\right)$ as the set composed of $U_{r}^{\left(j\right)}$ for all *j*. Each user *u* has a private input vector, $x_{u,j}$, for the *j*th secure additive aggregation; denote $x_{U^{\prime }}={\left\{x_{u,j}\;\mathrm{for}\mathrm{all}\;j\right\}}_{u\in U^{\prime }}$, where $U^{\prime }$ can be any subset of users. The same as the definition in the model statement, ${\mathrm{VIEW}}_{u}\left(x_{U}\right)$ represents the view of party u∈U∪{S} when the protocol runs with inputs $x_{U}$. Define C⊂U∪{S} as the set of semi-honest parties, and then ${\mathrm{VIEW}}_{C}\left(x_{U},U_{1},U_{2},U_{3},U_{4}\right)$ is the joint view of semi-honest adversaries during the entire protocol.

Firstly, we will consider the case in which semi-honest parties are a set of users, that is, C⊂U.

**Theorem** **6** (Security of the protocol $\Pi_{\mathrm{ReAgg}}$ against semi-honest users)**.** *There exists a probabilistic polynomial-time simulator, SIM, such that, for any security parameter λ, threshold parameter t, set of users U with |U|≥t, input vectors $x_{U}$, sets of online users $U⊇U_{1}⊇U_{2}^{\left(j\right)}⊇U_{3}^{\left(j\right)}⊇U_{4}^{\left(j\right)}$ for all j and set of semi-honest parties C⊆U with |C|≤t, the output of SIM is perfectly indistinguishable from ${\mathrm{VIEW}}_{C}$ as random variables; that is,*

$${\mathrm{VIEW}}_{C}\left(x_{U},U_{1},U_{2},U_{3},U_{4}\right)\equiv {\mathrm{SIM}}_{C}\left(x_{C},U_{1},U_{2},U_{3},U_{4}\right)$$

**Proof.**  For any user u∈C, all they have received during the entire protocol execution are public keys ${\left\{c_{v}^{pk},s_{v}^{pk}\right\}}_{v\in U_{1}\setminus \left\{u\right\}}$, ciphertexts ${\left\{e_{v,j,u}\right\}}_{v\in U_{2}^{\left(j\right)}\setminus \left\{u\right\}}$ for all *j* and a set of $U_{3}={\left\{U_{3}^{\left(j\right)}\right\}}_{j}$. Note that these messages do not depend on the inputs of the honest users. In fact, the only value sent by each honest user *v* that depend on their private input is $y_{v,j}$, which they sent to the server in Round 3 of the *j*th execution, but the server is honest. So, when knowing the inputs $x_{C}$ of user set C, the simulator SIM can be defined to use zero vectors for the inputs of all honest users and to use $x_{u,j}\left(u\in C\right)$ for the inputs of semi-honest users *u* in *j*th additive aggregation and then to simulate the execution of *j*th secure additive aggregation protocol. The simulator outputs the joint view of users in C in the simulation process, which is perfectly indistinguishable from ${\mathrm{VIEW}}_{C}$. □

When the server is semi-honest, it can share what it has received in the protocol execution with other semi-honest users. We show that the view of any such group of semi-honest parties can be simulated by a simulator, who is given the inputs of the users in that group and the final outputs of the server.

**Theorem** **7** (Security of protocol $\Pi_{\mathrm{ReAgg}}$ against semi-honest server and users)**.** *There exists a probability polynomial-time simulator, SIM, such that for any security parameter λ, threshold parameter t, set of users U with |U|≥t, input vectors $x_{U}$, sets of online users $U⊇U_{1}⊇U_{2}^{\left(j\right)}⊇U_{3}^{\left(j\right)}⊇U_{4}^{\left(j\right)}$ for all j and set of semi-honest parties C⊆U∪{S} with |C∖{S}|<t, the output of SIM is perfectly indistinguishable from ${\mathrm{VIEW}}_{C}$ as random variables; that is,*

$${\mathrm{VIEW}}_{C}\left(x_{U},U_{1},U_{2},U_{3},U_{4}\right)\equiv {\mathrm{SIM}}_{C}\left(x_{C},X={\left\{X_{j}\right\}}_{j},U_{1},U_{2},U_{3},U_{4}\right)$$

*where*

$$X_{j}=\left\{\begin{array}{c} \left(X_{j}^{1},\ldots ,X_{j}^{m}\right)\;\;\;\;\;\;\;\mathrm{if}\;\left|U_{3}^{\left(j\right)}\right|\geq \;t\\ ⊥\;\;\;\;\;\;\;\;\;\;\;\;\;\;\;\;\;\;\;\;\mathrm{otherwise} \end{array}\right.$$

The proof of the theorem is based on the security of Diffie–Hellman key agreement, the IND-CPA security of the authenticated encryption scheme, the security of secret sharing, the security of pseudorandom generator and the property of the bilinear map. It is given in Appendix F.

Finally, we show that the long-term keys of honest users remain safe during multiple aggregation executions. For the case where C⊆U with |C|<t, for any honest user u∈U, the adversary cannot reconstruct the one-time private key $s_{u,j}^{sk}$ for any *j*, since the number of shares it holds is less than the secret sharing threshold *t*. Therefore, the adversary learns nothing about the long-term private key $s_{u}^{sk}$. For the case where C⊆U with |C|=t, for any honest user u∈U, the adversary can reconstruct the one-time private key $s_{u,j}^{sk}=H{\left(j\right)}^{s_{u}^{sk}}$ for any *j*. According to the hardness assumption of a discrete logarithm, the adversary learns nothing about the long-term private key $s_{u}^{sk}$. For the case where the server is semi-honest, that is, C⊆U∪{S} with |C∖{S}|<t, for any honest user u∈U, the adversary can learn the one-time private key $s_{u,j}^{sk}=H{\left(j\right)}^{s_{u}^{sk}}$ only when $u\in U_{2}^{\left(j\right)}\setminus U_{3}^{\left(j\right)}$. According to the hardness assumption of the discrete logarithm, the adversary learns nothing about the long-term private key $s_{u}^{sk}$.

### 5.2. Key Reusable Secure Multiplicative Aggregation

Similarly, a key reusable secure multiplicative aggregation protocol can be constructed from the protocol $\Pi_{\mathrm{MulAgg}}$ using the idea of one-time private keys and the bilinearity of the bilinear map. In Appendix G, we provide a complete description of key reusable secure multiplicative protocol, together with its correctness and security proofs.

### 5.3. Discussion

The key reusable secure additive/multiplicative aggregation protocol allows users to reuse their public–private key pairs generated in Round 1, so it reduces the cost of broadcasting new public keys and conducting key agreement to obtain the keys of authenticated encryption in the multi-time secure additive/multiplicative aggregation process.

Moreover, all protocols we proposed can be further improved by changing their complete communication graph into a sparse *k*-regular communication graph [26], where k=O(logn). In this way, the overhead of polynomial magnitude for users and the server can be reduced to poly-logarithm.

## 6. Experimental

The performance evaluation of our proposed secure multiplication protocol was conducted using a Go implementation. The experimental testbed comprised one server node and two User nodes. The server was a Dell PowerEdge R760 (Dell, Round Rock, TX, USA) equipped with two Intel Xeon Platinum 8470 processors (52 cores/104 threads at 2.0 GHz) and 1024 GB of RAM. Both User nodes were identical Dell PowerEdge R750 systems (Dell, Round Rock, TX, USA), each configured with two Intel Xeon Gold 6338 CPUs (32 cores/64 threads at 2.0 GHz) and 256 GB of RAM. All machines ran the Ubuntu 22.04 operating system. The network connectivity between nodes was configured with a bandwidth of 80 Mb/s and a round-trip time (RTT) of 80 ms to simulate wide-area network conditions.

The experimental setup comprised the following parameters:

- **Key Agreement**: The Elliptic Curve Diffie–Hellman (ECDH) protocol was implemented using the NIST P-256 curve.
- **Hash Function**: SHA-256 was employed to hash the shared key.
- **Secret Sharing Scheme**: A t-out-of-n Shamir Secret Sharing scheme was utilized, where $t⩾\left\lfloor \frac{2n}{3}\right\rfloor +1$.
- **Authenticated Encryption**: AES-GCM with 128-bit keys was applied.
- **Pseudo-Random Number Generator**: AES-128 in counter mode was used.
- **Bilinear map**: Type A pairing implemented in the PBC library (https://pkg.go.dev/github.com/nik-u/pbc, accessed date 3 February 2026).
- **User Data**: Each user’s private data $X_{u}$ was represented as a vector of dimension *m*.
- **Module**: The modulus *R* is defined as the smallest prime greater than $2^{20}$, which is 1,048,583, and the modulus *q* is the smallest prime greater than $2^{40}$, namely 1,099,511,627,791.

### 6.1. Secure Multiplicative Aggregation Protocol $\Pi_{\;\mathrm{MulAgg}}$

To comprehensively analyze the performance of the secure multiplicative aggregation protocol $\Pi_{\mathrm{MulAgg}}$, we conducted experiments under two scenarios. All experimental data presented are the averages from ten experimental runs:

**Scenario** **1:** In this scenario, the size of the user input privacy data is fixed at 100KB while the number of users increases. We measured the average computation time overhead and communication overhead for each user and server, with the number of users set to {50,100,150,200,250,300} and user dropout rate to {0%,10%,20%,30%}.
**Scenario** **2:** In this scenario, the number of users was fixed at 300. We evaluated the average computation time and communication overhead for each user and the server by varying the size of privacy-sensitive data inputs. The input sizes were set to {10KB,50KB,100KB,150KB,200KB,250KB}, and the user dropout rates were set to {0%,10%,20%,30%}.

**Figure 5:**
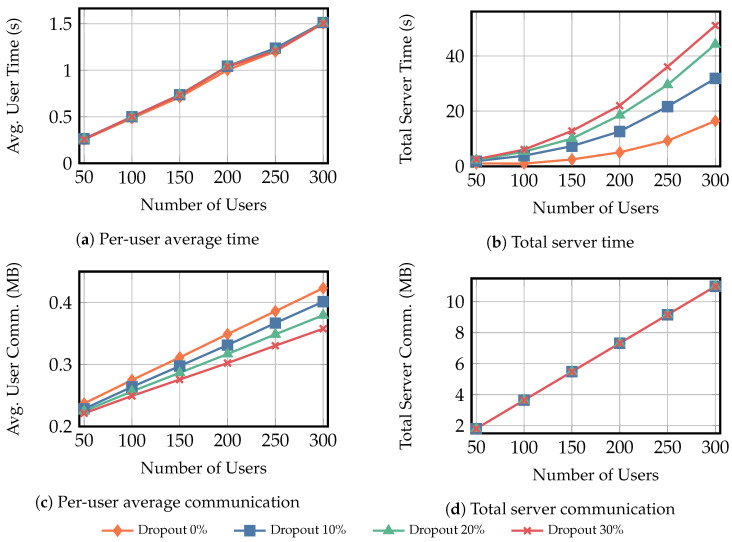
Scenario 1 results for fixed user private-data size $X_{u}=100\;\mathrm{KB}$, where the number of users varies from 50 to 300, and the dropout rate varies over {0%,10%,20%,30%}. Subfigures (**a**,**c**) show the average per-user computation time and communication overhead, while (**b**,**d**) show the total server computation time and communication overhead. “Avg.” denotes average and “Comm.” denotes communication.

Figure 5 shows the experimental results for scenario 1. Figure 5a,b report the average per-user computation time and the total server computation time, respectively. As the number of users increases, the average computation time per user increases accordingly and is only slightly affected by the dropout rate. By contrast, the total computation time of the server increases significantly as the dropout rate rises, because a higher dropout rate requires the server to recover the private keys of more dropped-out users. Figure 5c,d report the average per-user communication overhead and the total server communication overhead, respectively. Both quantities increase approximately linearly with the number of users. In addition, a higher dropout rate leads to lower average communication overhead per user, while the total communication overhead of the server remains almost unaffected by the dropout rate.

**Figure 6:**
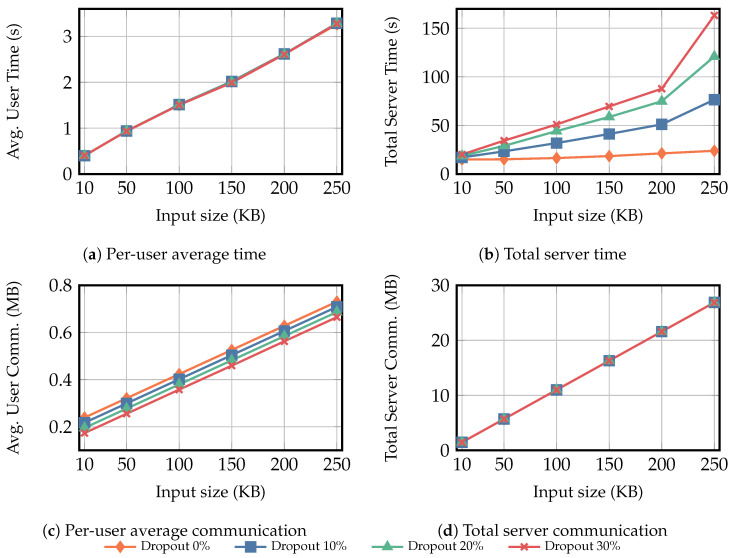
Scenario 2 results with a fixed number of users (*N* = 300). The input size $X_{u}$ varies over {10KB,50KB,100KB,150KB,200KB,250KB}, and the dropout rate varies over {0%,10%,20%,30%}. Subfigures (**a**,**c**) report the average per-user computation time and communication overhead, while (**b**,**d**) report the total computation time and communication overhead of the server. “Avg.” denotes average, and “Comm.” denotes communication.

In Scenario 2, we evaluate how varying the per-user private input size affects the computation and communication overhead of users and the server. The results are shown in Figure 6; the overall trends are consistent with those observed in Scenario 1.

Figure 5b and Figure 6b show that, in both scenarios, the total server computation time grows more rapidly under higher dropout rates. In Scenario 1, as the number of users grows from 50 to 300, the server time increases by 15.32 times, 15.92 times, 18.71 times, and 19.11 times under dropout rates {0%,10%,20%,30%}, respectively. In Scenario 2, when the per-user private input increases from 10KB to 250KB, the corresponding growth factors are 1.60 times, 4.49 times, 6.50 times, and 8.14 times, respectively. This growth is dominated by Round 4, where the main cost comes from ${\mathrm{PRG}}_{1}\left(\cdot \right)$ and ${\mathrm{PRG}}_{2}\left(\cdot \right)$ for mask recovery. Although higher dropout reduces the number of $t_{v}$ and $k_{v}$ computations, the server must spend substantially more effort reconstructing $s_{v}^{sk}$ and derive $t_{v,u}$ and $k_{v,u}$ via ${\mathrm{PRG}}_{1}\left(\cdot \right)$ and ${\mathrm{PRG}}_{2}\left(\cdot \right)$, thereby increasing the total server overhead.

Figure 5a and Figure 6a show that the average per-user computation time is largely insensitive to the dropout rate. In Scenario 1, increasing the number of users from 50 to 300 increases the average per-user computation time by 4.85 times, 4.75 times, 4.82 times, and 4.81 times under dropout rates of {0%,10%,20%,30%}, respectively. In Scenario 2, increasing the per-user private input from 10KB to 250KB increases the average per-user computation time by 8.11 times, 8.26 times, 8.17 times, and 8.16 times, respectively. Rounds 1–3 incur fixed-cost operations (key generation, ciphertext computation, and masked-data generation), while Round 4 decryption is performed only by online users; thus, higher dropout slightly increases Round 4 time but has a negligible impact on the total. Overall, Round 3 dominates the average user time, mainly due to ${\mathrm{PRG}}_{1}\left(\cdot \right)$ and ${\mathrm{PRG}}_{2}\left(\cdot \right)$ for mask generation, followed by computing the obfuscated private data.

Figure 5c,d and Figure 6c,d exhibit similar communication patterns in both scenarios. The average per-user communication overhead grows nearly linearly with the number of users or the input size, and decreases slightly as the dropout rate increases. In contrast, the total server communication overhead is largely insensitive to the dropout rate and is mainly determined by the number of users and the input size.

### 6.2. Key Reusable Secure Additive Aggregation $\Pi_{\;\mathrm{ReAgg}}$

To evaluate the performance of the reusable secure additive aggregation protocol $\Pi_{\mathrm{ReAgg}}$, experiments were conducted under two scenarios, as follows. In the second scenario, we implemented the non-reusable secure additive aggregation protocol from [24] and compared it on the same server. All results represent the average of ten independent runs.

**Scenario** **1:** Each user’s private input was fixed at 100KB. The number of users ranged from 100 to 500 in increments of 50, under dropout rates of {0%,10%,20%,30%}. We measured the average computation time and communication overhead per-user and server.
**Scenario** **2:** In this scenario, the number of users is fixed at 100, with each input again sized at 100KB. Dropout rates were set to {0%,10%,20%,30%}. We conducted a comparative experiment between reusable and non-reusable additive aggregation protocols, with each protocol performing q={1,⋯,10} secure aggregation iterations. The reusable protocol executes Round 1 only once in the first iteration (i.e., q=1) and reuses subsequent Round 2–4 in the remaining iterations (i.e., q={2,⋯,10}), whereas the non-reusable protocol repeats the full protocol each iteration. We also evaluated the average computation time and communication overhead for per-user and server.

**Figure 7:**
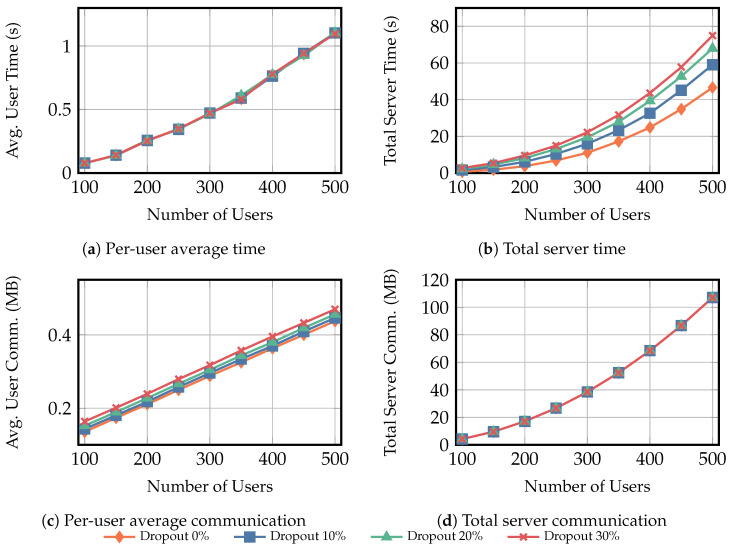
Scenario 1 results for fixed user private-data size $X_{u}=100\;\mathrm{KB}$, where the number of users varies from 100 to 500, and the dropout rate varies over {0%,10%,20%,30%}. Subfigures (**a**,**c**) report the average per-user computation time and communication overhead, while (**b**,**d**) report the total computation time and communication overhead of the server. “Avg.” denotes average and “Comm.” denotes communication.

Figure 7a shows that the average per-user computation time increases monotonically with the number of users *K* and is almost unaffected by the dropout rate. When *K* increases from 100 to 500, the average per-user computation time increases by 14.47 times, 14.19 times, 14.30 times, and 14.25 times under dropout rates of 0%, 10%, 20%, and 30%, respectively. In contrast, Figure 7b indicates that the server computation overhead is sensitive to the dropout rate: at K=100 the total server time increases to 2.36 times, 3.32 times, and 4.04 times that of the no-dropout case. This consistent gap indicates that user dropouts mainly introduce additional recovery and reconstruction work on the server side, whereas the user-side cost remains dominated by fixed per-round operations.

Figure 7c reports the average per-user communication overhead. It increases with *K* and also increases mildly with the dropout rate. For instance, at K=500, the average per-user communication overhead under a dropout rate of 30% is 1.07 times that of the no-dropout case. Figure 7d shows that the server total communication grows rapidly with *K*, when *K* increases from 100 to 500, it grows by about 25.16 times while remaining almost unaffected by the dropout rate. At K=500, the total server communication overhead under a dropout rate of 30% is essentially 1.00 times that of the no-dropout case. A round-level breakdown further shows that the server communication is dominated by the Round 2 broadcast of aggregated ciphertexts; for example, at K=500, the Round 2 communication accounts for about 1.00 times the total server communication overhead, whereas the Round 1 key broadcast and the Round 3 UserNameList contribute only a negligible fraction of the total. These results indicate that dropouts mainly impact the server computation overhead, rather than the total communication overhead.

**Figure 8:**
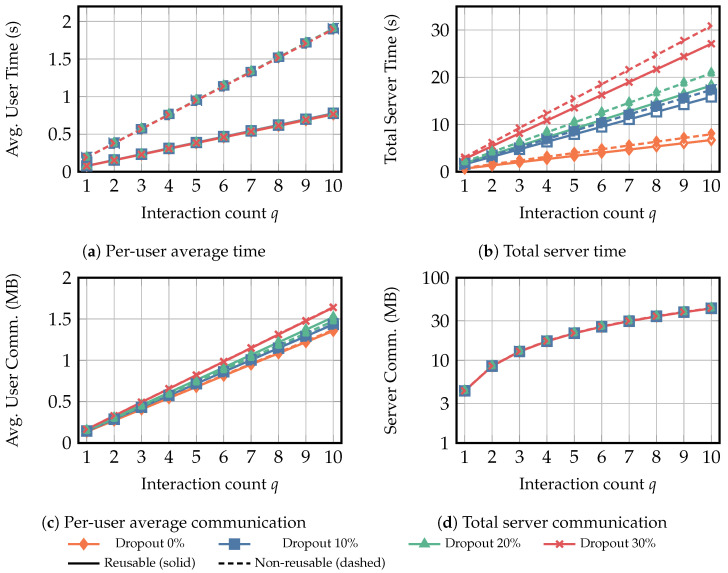
Scenario 2 results for fixed user private-data size $X_{u}=100\;\mathrm{KB}$ and a fixed number of users (N=100), where the interaction count *q* varies from 1 to 10, and the dropout rate varies over {0%,10%,20%,30%}. The figure compares the reusable and non-reusable additive aggregation protocols. Subfigures (**a**,**c**) report the average per-user computation time and communication overhead, while (**b**,**d**) report the total computation time and communication overhead of the server. “Avg.” denotes average, and “Comm.” denotes communication.

Figure 8a,b report the average cumulative computation time of the user and the server when the input size is fixed at 100 KB and *q* increases from 1 to 10. For the user side, the computation time is largely insensitive to the dropout rate. As *q* increases, the gap between the reusable and non-reusable schemes widens, because the non-reusable scheme scales approximately linearly with *q*, whereas the reusable scheme amortizes Round 1 over repeated interactions. For example, at q=10, the average per-user computation time of the reusable scheme is about 0.40 times and 0.41 times that of the non-reusable scheme under dropout rates of 0% and 30%, respectively. In contrast, the total server computation time is clearly sensitive to the dropout rate in both schemes. Under the non-reusable scheme, the server computation time at q=1 increases to 2.18 times, 2.63 times, and 3.89 times, when q=10 the corresponding factors remain 2.18 times, 2.63 times, and 3.89 times. The reusable scheme consistently reduces the total server computation time but less significantly than on the user side. At q=10, it reduces the total server computation time by about 16% and 12% compared with the non-reusable scheme under dropout rates of 0% and 30%, respectively. This indicates that the server-side cost is still dominated by the non-reused rounds and the dropout-driven recovery operations.

Figure 8c,d report the cumulative communication overhead for the user and the server. The average per-user communication overhead grows close to linearly with *q* and increases with the dropout rate. Concretely, under the non-reusable scheme, when q=10, the average per-user communication overhead at a dropout rate of 30% is about 1.20 times that at a dropout rate of 0%. Comparing the two schemes reveals that the reusable and non-reusable curves remain very close. For example, at q=10, the average per-user communication overhead of the reusable scheme is reduced to 0.99 times and 1.00 times that of the non-reusable scheme under dropout rates of 0% and 30%, respectively, indicating that Round 1 reuse provides only marginal communication savings on the user side. On the server side, the communication overhead curves for the reusable and non-reusable schemes are nearly indistinguishable over q=1,…,10, and the gap increases only slightly with *q*. Under the non-reusable scheme, when q=10, the total server communication overhead at a dropout rate of 30% is essentially 1.00 times that at a dropout rate of 0%, showing that the server communication overhead is almost unaffected by the dropout rate. Moreover, the reusable scheme provides only a marginal reduction in total server communication overhead. For instance, at q=10 and a dropout rate of 0%, the total server communication overhead of the reusable scheme is reduced to 0.99 times that of the non-reusable scheme. This indicates that reusing Round 1 has a limited impact on communication overhead, and that the overall communication cost is mainly determined by the non-reusable rounds.

### 6.3. Practical Implications and Applicability

While the present work is primarily a cryptographic and protocol-level contribution, its evaluated parameter regimes are also relevant to practical federated learning deployments. The considered user scale K∈[100,500] is representative of the number of clients often sampled in one federated learning round from a much larger population, especially in cross-device settings. Similarly, private input sizes ranging from tens to hundreds of kilobytes are consistent with compressed model updates, partial gradient vectors, or feature statistics by mobile devices and edge clients. The dropout regimes considered in our experiments, from 0% to 30%, also match realistic federated learning conditions, where client availability is affected by device mobility, battery level, network instability, and intermittent participation. Under such conditions, our experimental results suggest that the protocol remains practical on the user side, while the main computational burden caused by dropouts is concentrated at the server, which is consistent with the resource asymmetry between mobile clients and aggregation servers.

The proposed multiplicative aggregation protocol is particularly beneficial when the target quantity is multiplicative in nature or depends on interaction terms that cannot be captured by standard secure summation alone. In such cases, the goal is not simply to securely sum local updates but to privately compute product-type statistics or interaction-dependent quantities across users. Typical examples include multiplicative feature interactions, product-based statistics, geometric-mean-style aggregates, likelihood-related terms, and privacy-preserving analytics in which correlations or joint effects among local inputs are important. From this perspective, multiplicative aggregation should be viewed not as a replacement for conventional secure summation, but as a complementary primitive that enables a richer class of privacy-preserving computations beyond additive aggregation.

The advantage of key reuse becomes more pronounced in repeated-training or repeated-query settings, which are typical in federated learning. In practical federated learning systems, the same client cohort may participate in multiple consecutive rounds, local retraining sessions, or repeated secure analytics tasks over related data distributions. In such cases, amortizing the one-time cost of Round 1 across repeated interactions can significantly reduce cumulative computation, especially on the user side, as confirmed by our experiments. Therefore, the practical benefit of reuse is strongest in long-running federated learning with repeated rounds and relatively stable participation patterns.

## 7. Conclusions

In this paper, we have proposed a secure multiplicative aggregation protocol. The protocol defines the interaction between the server and users who each have a high-dimensional private vector, and finally, the server outputs the component-wise product of online users’ input vectors. The execution process guarantees that no private information of honest users will be revealed. Users are allowed to drop out at any time of the protocol execution, and constant rounds of the protocol can remove the dropout users’ information from the product. We have proven that the protocol is correct and secure against semi-honest adversaries under correctness and security parameters.

We also discussed the case in which a fixed set of users want to perform secure additive/multiplicative aggregation together many times. By using one-time private keys for each aggregation execution and the bilinearity of a bilinear map, we constructed a key reusable secure additive aggregation protocol and a key reusable secure multiplicative aggregation protocol. All three protocols we presented have an overhead of polynomial magnitude with regard to the number of users *n* and the length of private vectors *m* by theoretical analysis.

It can be noted that we have only considered secure aggregation in the semi-honest security model, and a trusted authority is needed in the setup phase of our protocols. We will leave the construction of a malicious secure multiplicative aggregation protocol for future work.

Finally, we implement the secure multiplicative aggregation protocol and the key-reusable secure additive aggregation protocol and KB, increasing the number of users from K=100 to 500, the average per-user computation time increases by about 14.2–14.5 times, while the total server computation time increases by about 27.7–69.5 times, depending on the dropout rate. When the dropout rate increases from 0% to 30% at K=500, the total server computation time increases by about 1.61 times, whereas the average per-user computation time remains essentially unchanged. From the communication perspective, increasing *K* from 100 to 500 enlarges the average per-user communication overhead by about 2.9–3.2 times while leaving the total server communication overhead almost unaffected by the dropout rate. Moreover, across repeated interactions q∈{1,…,10}, reusing Round 1 reduces the cumulative user and server computation time by about 2.49 times and 1.18 times at q=10, respectively, while the cumulative communication overhead changes only marginally, indicating that communication is dominated by the non-reused rounds.

## Figures and Tables

**Table 1:** Summary of parameters used throughout the protocols.

Parameters	Description
*n*	the number of online users at the beginning of protocol execution
*t*	threshold parameter of secret sharing
λ	security parameter
η	correctness parameter
$Z_{R}^{m}$	the space of users’ private input vectors
$Z_{p}$	the field of secret sharing, the space of secret keys in key agreement scheme
$Z_{q}$	the space of auxiliary vectors (which are used as inputs in secure aggregation)
G	a cyclic group with order *p* and a generator *g*, the space of public keys in key agreement scheme
$G_{T}$	a cyclic group with order *p* and a generator $g_{T}$, the output space of bilinear map
*U*	the set of original users
$U_{i}$	the set of users whose message has been received by the server in *i*th round of the aggregation protocol
$U_{i}^{\left(j\right)}$	the set of users whose message has been received by the server in *i*th round of *j*th secure additive/multiplicative aggregation protocol

## Data Availability

The original contributions presented in this study are included in the article. Further inquiries can be directed to the corresponding author.

## Appendix A. Preliminaries

### Appendix A.1. Authenticated Encryption

If one party wants to send a secret message to another party, but the communication between them has to go through the third party that is not trustable, authenticated encryption can guarantee the confidentiality and integrity for the message exchanged between the two parties. The party who sends secret message is called the sender, and the one who receives message is called the receiver.

An (symmetric) authenticated encryption scheme consists of three algorithms, the key generation algorithm, the encryption algorithm and the decryption algorithm. The key generation algorithm AE.gen(λ)→k takes as input security parameter λ, and outputs a private key, *k*, that is known to the sender and receiver. Without a loss of generality, it may be assumed that key *k* is sampled as uniformly random strings. The encryption algorithm AE.enc(k,m)→c run by the sender takes as input a key and a message, and outputs a ciphertext. The decryption algorithm AE.dec(k,c)→m run by the receiver takes as input a key and a ciphertext they received from the sender, and outputs a message *m* in the plaintext space or a special error symbol ⊥.

The correctness of the scheme requires that, for any key, $k\in {\left\{0,1\right\}}^{\lambda }$, any message *m* in the plaintext field, we have AE.dec(k,AE.enc(k,m))=m.

The security of the scheme requires indistinguishability under a chosen plaintext attack (IND-CPA) [28].

**Definition** **A1.**
*Consider a probabilistic experiment below, where A is a probabilistic polynomial-time algorithm, b is a randomly chosen bit, Π=(gen,enc,dec) is a probabilistic encryption scheme, and λ is the security parameter.*

*Chosen plaintext attack indistinguishability experiment ${CPA-EXP}_{A,\Pi }\left(\lambda \right)$:*
*1.* k←gen(λ)*.* *2. Algorithm* A *(adversary) has access to an encryption with key k oracle* enc(k,·) *for free in the experiment. That is,* A *can always choose a message m from plaintext field, and use the oracle to obtain the ciphertext* enc(k,m)*.* *3. Adversary* A *chooses a pair of messages* $m_{0},m_{1}$ *in the plaintext field that satisfy* $|m_{0}\left|=\right|m_{1}|$ *and sends them to a scheme* Π *simulator with k.* *4. The simulator computes* $c\leftarrow \mathrm{enc}(k,m_{b})$ *(c is called the challenge ciphertext) and gives it to* A*.* *5.* $A\left(c,m_{0},m_{1},\mathrm{enc}\left(k,\cdot \right)\right)\to b^{\prime }$*.* *6. If* $b=b^{\prime }$*, outputs 1; if* $b\neq b^{\prime }$*, outputs 0.* *An encryption scheme,* Π=(gen,enc,dec)*, is called indistinguishable against chosen plaintext attacks if, for any probabilistic polynomial-time algorithm,* A*, there exists a negligible function,* negl*, such that*

$$|\mathrm{Pr}\left[{\mathrm{CPA-EXP}}_{A,\Pi }\left(\lambda \right)=1\right]-\frac{1}{2}|\;\leq negl\left(\lambda \right)$$

That is to say, in the case that has access to an encryption oracle associated with some key, *k*, but the key *k* is kept unknown, that two different messages encrypted by key *k* are indistinguishable.

### Appendix A.2. Pseudorandom Generator

What we need are two secure pseudorandom generators, ${\mathrm{PRG}}_{1}$ and ${\mathrm{PRG}}_{2}$; they are variants of the generic pseudorandom generator [29]. Specifically, ${\mathrm{PRG}}_{1}$ takes as input a uniform random seed from a finite cyclic group, and its output space is ${\left(Z_{R}^{*}\right)}^{m}$. ${\mathrm{PRG}}_{2}$ takes as input a uniform random seed from a finite cyclic group, and its output space is $Z_{q}^{m}$. Here, *R* and *q* are two primes, such that $Z_{R}^{m}$ is the space of users’ private input vector, and $Z_{q}$ is the field for secret sharing.

Security for a pseudorandom generator guarantees that its output on a uniformly random seed is computationally indistinguishable from a uniformly sampled element of the output space, as long as the seed is hidden from the distinguisher.

## Appendix B. Lemmas Used in Security Proofs

**Lemma** **A1.**
*Given prime q and positive integer m, for any fixed set V, a family of vectors ${\left\{z_{v}\right\}}_{v\in V}$ with $\forall v\in V,z_{v}\in Z_{q}^{m}$ holds that*

$$\left\{{\left\{p_{u,v}\leftarrow Z_{q}^{m}\right\}}_{u<v},p_{u,v}=-p_{v,u}\;\forall u>v\;:\{z_{u}+\sum_{v\in V\setminus \{u\}}p_{u,v}\;\;\left(\mathrm{mod}\;q\right){\}}_{u\in V}\right\}$$

$$\equiv \{{\left\{d_{u}\leftarrow Z_{q}^{m}\right\}}_{u\in V}\;s.t.\;\sum_{u\in V}d_{u}=\sum_{u\in V}z_{u}\;\;\left(\mathrm{mod}\;q\right)\;:{\left\{d_{u}\right\}}_{u\in V}\}$$

*where ≡ represents that two random variables are identically distributed.*

**Proof.** Prove by induction on the number of elements *V* contains. For the case in which |V|=1 and |V|=2, the equation holds obviously. Assume that the conclusion is right for |V|=k; we will prove it also holds for |V|=k+1. Fix w∈V, for any u∈V∖{w}, and define $\delta_{u}=p_{u,w}$. By the induction hypothesis,

$$\left\{{\left\{p_{u,v}\leftarrow Z_{q}^{m}\right\}}_{u<v},p_{u,v}=-p_{v,u}\;\forall u>v\in V\setminus \left\{w\right\}\;:\{\left(z_{u}+\delta_{u}\right)+\sum_{v\in V\setminus \{u,w\}}p_{u,v}\;\;\left(mod\;q\right){\}}_{u\in V\setminus \{w\}}\right\}$$

$$\equiv \{{\left\{d_{u}\leftarrow Z_{q}^{m}\right\}}_{u\in V\setminus \{w\}}\;s.t.\;\sum_{u\in V\setminus \{w\}}d_{u}=\sum_{u\in V\setminus \{w\}}\left(z_{u}+\delta_{u}\right)\;\;\left(\mathrm{mod}\;q\right)\;:{\left\{d_{u}\right\}}_{u\in V\setminus \{w\}}\}$$

$$\equiv \{{\left\{d_{u}\leftarrow Z_{q}^{m}\right\}}_{u\in V\setminus \{w\}}\;s.t.\;\sum_{u\in V\setminus \{w\}}d_{u}=\sum_{u\in V\setminus \{w\}}z_{u}+\sum_{u\in V\setminus \{w\}}p_{u,w}\;\;\left(\mathrm{mod}\;q\right)\;:{\left\{d_{u}\right\}}_{u\in V\setminus \{w\}}\}$$

Let

$$d_{w}=z_{w}+\sum_{u\in V\setminus \{w\}}p_{w,u}=z_{w}-\sum_{u\in V\setminus \{w\}}p_{u,w}\;\;\left(\mathrm{mod}\;q\right)$$

Since $p_{u,w}$ is randomly and uniformly chosen, $d_{w}$ is randomly and uniformly distributed in $Z_{q}^{m}$ and satisfies

$$\sum_{u\in V}d_{u}=\sum_{u\in V}z_{u}$$

□

**Lemma** **A2.**
*Given prime R and positive integer m, for any fixed set V, a family of vectors ${\left\{{\tilde{x}}_{v}\right\}}_{v\in V}$ with $\forall v\in V,{\tilde{x}}_{v}\in {\left(Z_{R}^{*}\right)}^{m}$ holds that*

$$\left\{{\left\{p_{u,v}\leftarrow {\left(Z_{R}^{*}\right)}^{m}\right\}}_{u<v},p_{u,v}=p_{v,u}^{-1}\;\;\left(\mathrm{mod}\;R\right)\;\forall u>v\;:\{{\tilde{x}}_{u}\cdot \prod_{v\in V\setminus \{u\}}p_{u,v}\;\;\left(\mathrm{mod}\;R\right){\}}_{u\in V}\right\}$$

$$\equiv \{{\left\{w_{u}\leftarrow {\left(Z_{R}^{*}\right)}^{m}\right\}}_{u\in V}\;s.t.\;\prod_{u\in V}w_{u}=\prod_{u\in V}{\tilde{x}}_{u}\;\;\left(\mathrm{mod}\;R\right)\;:{\left\{w_{u}\right\}}_{u\in V}\}$$

*where ≡ represents the fact that two random variables are identically distributed.*

**Proof.** Note that, when |V|=2, for a fixed ${\tilde{x}}_{u}\in {\left(Z_{R}^{*}\right)}^{m}$, it holds that

$$\left\{p_{u,v}\leftarrow {\left(Z_{R}^{*}\right)}^{m}\;:{\tilde{x}}_{u}\cdot p_{u,v}\right\}\equiv \left\{w_{u}\leftarrow {\left(Z_{R}^{*}\right)}^{m}\;:w_{u}\right\}$$

The following reduction step is similar to Lemma 1. □

## Appendix C. Proof of Theorem 2

**Proof.** First, show that the server has got the correct value of the sum and product at the 4th step of Round 4.

$$\begin{array}{cc} (1)\mathrm{LHS} & =\sum_{u\in U_{3}}l_{u}-\sum_{u\in U_{3}}k_{u}+\sum_{u\in U_{3},v\in U_{2}\setminus U_{3},v>u}k_{v,u}-\sum_{u\in U_{3},v\in U_{2}\setminus U_{3},v<u}k_{v,u}\;\;\left(\mathrm{mod}\;q\right)\\ \; & =(\sum_{u\in U_{3}}z_{u}+\sum_{u\in U_{3}}k_{u}+\sum_{u\in U_{3},v\in U_{2},u>v}k_{u,v}-\sum_{u\in U_{3},v\in U_{2},u<v}k_{u,v})\\ \; & \;-\sum_{u\in U_{3}}k_{u}+\sum_{u\in U_{3},v\in U_{2}\setminus U_{3},v>u}k_{v,u}-\sum_{u\in U_{3},v\in U_{2}\setminus U_{3},v<u}k_{v,u}\;\;\left(\mathrm{mod}\;q\right)\\ \; & =\sum_{u\in U_{3}}z_{u}+\left(\sum_{u\in U_{3},v\in U_{2},u>v}k_{u,v}-\sum_{u\in U_{3},v\in U_{2}\setminus U_{3},v<u}k_{v,u}\right)\\ \; & \;-\left(\sum_{u\in U_{3},v\in U_{2},u<v}k_{u,v}-\sum_{u\in U_{3},v\in U_{2}\setminus U_{3},v>u}k_{v,u}\right)\;\;\left(\mathrm{mod}\;q\right)\\ \; & =\sum_{u\in U_{3}}z_{u}+\sum_{u\in U_{3},v\in U_{3},u>v}k_{u,v}-\sum_{u\in U_{3},v\in U_{3},u<v}k_{u,v}\;\;\left(\mathrm{mod}\;q\right)\\ \; & =\sum_{u\in U_{3}}z_{u}\;\;\left(\mathrm{mod}\;q\right)=\left(1\right)\mathrm{RHS} \end{array}$$

It is similar to show that (2) holds. So, the server obtains $\sum_{u\in U_{3}}z_{u}$ and $\prod_{u\in U_{3}}{\tilde{x}}_{u}$ correctly. Consider the set

$$J=\{j\in \left[m\right]|x_{u}^{j}\neq 0\;\mathrm{holds}\mathrm{for}\mathrm{all}\;u\in U_{3}\}$$

Due to the definition of $z_{u}$ and ${\tilde{x}}_{u}$ in the Setup Round, it holds that $z_{u}^{j}=0$ for every $j\in J,u\in U_{3}$; that is, I∩J=⌀. It also holds that $x_{u}^{j}={\tilde{x}}_{u}^{j}$ for every $j\in J,u\in U_{3}$. Multiply for all $u\in U_{3}$, and we get

$$\prod_{u\in U_{3}}x_{u}^{j}=\prod_{u\in U_{3}}{\tilde{x}}_{u}^{j}=X^{j}\;\;\left(\mathrm{mod}\;R\right)$$

So, the *j*th (∀j∈J) component of the output vector is the correct product result. Note that $\prod_{u\in U_{3}}x_{u}^{j}=0$ holds for every j∈[m]∖J; therefore, if the output is incorrect, the only case that may happen is that the *j*th component of the output vector is nonzero for some j∈[m]∖J. For any fixed j∈[m]∖J, consider the set

$$U^{\left(j\right)}=\left\{u\in U_{3}|x_{u}^{j}=0\right\}$$

Every user in this set randomly chooses an element from $Z_{q}\setminus \left\{0\right\}$ as $z_{u}^{j}$; these selections are independent of each other. Define the event $E_{j}$ to be $\sum_{u\in U_{3}}x_{u}^{j}=\sum_{u\in U^{\left(j\right)}}x_{u}^{j}=0\;\;\left(\mathrm{mod}\;q\right)$; then, this event will happen with probability $\frac{1}{q-1}$. If event $E_{j}$ does not happen, then we have j∈I by the protocol definition, so $X^{j}=0$, the *j*th component of the output vector is correct. The probability $P_{error}$ that the protocol outputs incorrectly is exactly the probability that event $E_{j}$ happens for some j∈[m]∖J. By Boole’s inequality,

$$\begin{array}{cc} P_{error} & =\mathrm{Pr}[\exists j\in \left[m\right]\setminus J\;\mathrm{such}\mathrm{that}\;E_{j}]\\ \; & \leq \sum_{j\in [m]\setminus J}\mathrm{Pr}\left[E_{j}\right]\\ \; & \leq \frac{m}{q-1}<\frac{1}{2^{\eta }} \end{array}$$

So, the protocol $\Pi_{\mathrm{MulAgg}}$ has the correct output with probability at least $1-\frac{1}{2^{\eta }}$. □

## Appendix D. Proof of Theorem 4

**Proof.** First, discuss the case in which $|U_{3}|\geq t$. We will define a simulator SIM and its outputs through a series of subsequent modification to the random variable ${\mathrm{VIEW}}_{C}$, so that any two subsequent random variables are computationally indistinguishable.

${\mathrm{Hyb}}_{0}$

This random variable is distributed exactly as ${\mathrm{VIEW}}_{C}$, the joint view of semi-honest parties C in a real execution of the protocol.

${\mathrm{Hyb}}_{1}$

Compare to ${\mathrm{Hyb}}_{0}$; in this hybrid, we change the behavior of simulated honest parties in the set $U_{2}\setminus C$. When any user $u\in U_{2}\setminus C$ sends encrypted secret shares $e_{u,v}$ to any user $v\in U_{2}\setminus C$, and also when *v* decrypts this ciphertext (if they are still online in Round 4), instead of using $\mathrm{KA}.\mathrm{agree}(c_{u}^{sk},c_{v}^{pk})$ to encrypt and decrypt, they use a uniformly random encryption key, $c_{u,v}=c_{v,u}$, sampled by the simulator. The security of Diffie–Hellman key agreement guarantees that this hybrid is indistinguishable from the previous one.

${\mathrm{Hyb}}_{2}$

Compare to ${\mathrm{Hyb}}_{1}$; in this hybrid, when any user $u\in U_{2}\setminus C$ sends encrypted secret shares to any user $v\in U_{2}\setminus C$, instead of sending the encryptions of the shares of $s_{u}^{sk}$ and $b_{u}$ corresponding to *v*, user *u* sends the encryption of 0 (with appropriate length) to user *v* via a server. However, when *v* decrypts the ciphertext (if they are still online in Round 4), they continue to respond with the correct shares of $s_{u}^{sk}$ and $b_{u}$. Since the key remains same and only the contents of the ciphertexts have changed, the IND-CPA security of the authenticated encryption scheme guarantees that this hybrid is indistinguishable from the previous one.

${\mathrm{Hyb}}_{3}$

Define $U^{*}=U_{2}\setminus U_{3}$. Compare to ${\mathrm{Hyb}}_{2}$; in this hybrid, when any user $u\in U^{*}$ sends encrypted secret shares to any user v∈C, instead of sending the encryptions of the shares of $s_{u}^{sk}$ and $b_{u}$ corresponding to *v*, user *u* sends the encryption of the shares of $s_{u}^{sk}$ and 0 (using a different sharing polynomial to share 0 for every $u\in U^{*}$) corresponding to *v* to user *v* via server. Since user $u\in U^{*}$ has dropped out before they have successfully sent messages associated to their private input to the server, according to the protocol, semi-honest parties cannot get the shares of $b_{u}$ corresponding to any honest party in Round 4, so there are only |C∖{S}|<t shares of $b_{u}$ contained in all the messages that the simulator has sent and received. The security of secret sharing guarantees that this hybrid is indistinguishable from the previous one.

${\mathrm{Hyb}}_{4}$

Compare to ${\mathrm{Hyb}}_{3}$; in this hybrid, when any user $u\in U_{3}\setminus C$ sends encrypted secret shares to any user v∈C, instead of sending the encryptions of the shares of $s_{u}^{sk}$ and $b_{u}$ corresponding to *v*, user *u* sends the encryption of the shares of 0 (using a different sharing polynomial to share 0 for every $u\in U_{3}\setminus C$) and $b_{u}$ corresponding to *v* to user *v* via server. Since user $u\in U_{3}\setminus C$ has successfully sent messages associated with their private input to the server, according to the protocol, semi-honest parties cannot get the shares of $s_{u}^{sk}$ corresponding to any honest party in Round 4, so there are only |C∖{S}|<t shares of $s_{u}^{sk}$ contained in all the messages that the simulator has sent and received. The security of secret sharing guarantees that this hybrid is indistinguishable from the previous one.

${\mathrm{Hyb}}_{5}$

Compare to ${\mathrm{Hyb}}_{4}$; in this hybrid, when any user $u\in U_{3}\setminus C$ computes the seed $s_{u,v}$ corresponding to user $v\in U_{3}\setminus C$ in Round 3, instead of using $\mathrm{KA}.\mathrm{agree}(s_{u}^{sk},s_{v}^{pk})$ as the seed of cancellable masks, user *u* expands $s_{u,v}=s_{v,u}\in G$, which is uniformly and randomly sampled by the simulator to obtain masks $t_{u,v}$ and $k_{u,v}$. The security of the Diffie–Hellman key agreement guarantees that this hybrid is indistinguishable from the previous one.

${\mathrm{Hyb}}_{6}$

Compare to ${\mathrm{Hyb}}_{5}$; in this hybrid, when any user $u\in U_{3}\setminus C$ computes the masks $t_{u,v}$ and $k_{u,v}$ corresponding to user $v\in U_{3}\setminus C$ in Round 3, instead of using pseudorandom generators to get $t_{u,v}={\mathrm{PRG}}_{1}\left(s_{u,v}\right)$ and $k_{u,v}={\mathrm{PRG}}_{2}\left(s_{u,v}\right)$, user *u* takes $t_{u,v}=t_{v,u}\leftarrow {\left(Z_{R}^{*}\right)}^{m}$ and $k_{u,v}=k_{v,u}\leftarrow Z_{q}^{m}$ (with appropriate length) sampled uniformly and randomly by the simulator as masks. In this hybrid, we substitute the output of a PRG on a randomly generated seed with a uniformly value on the output space; the security of the pseudorandom generator guarantees that this hybrid is indistinguishable from the previous one.

${\mathrm{Hyb}}_{7}$

Compare to ${\mathrm{Hyb}}_{6}$; in this hybrid, when any user $u\in U_{3}\setminus C$ computes the masked input vector in Round 3, instead of computing

$$\begin{array}{cc} y_{u} & ={\tilde{x}}_{u}\cdot t_{u}\cdot \prod_{v\in U_{2},u>v}t_{u,v}\cdot \prod_{v\in U_{2},u<v}t_{u,v}^{-1}\;\;\left(\mathrm{mod}\;R\right)\\ \; & ={\tilde{x}}_{u}\cdot t_{u}\cdot \prod_{v\in U_{3}\setminus C,u>v}t_{u,v}\cdot \prod_{v\in U_{3}\setminus C,u<v}t_{u,v}^{-1}\cdot \prod_{v\in U_{2}\setminus \left(U_{3}\setminus C\right),u>v}t_{u,v}\cdot \prod_{v\in U_{2}\setminus \left(U_{3}\setminus C\right),u<v}t_{u,v}^{-1}\;\;\left(\mathrm{mod}\;R\right) \end{array}$$

$$\begin{array}{cc} l_{u} & =z_{u}+k_{u}+\sum_{v\in U_{2},u>v}k_{u,v}-\sum_{v\in U_{2},u<v}k_{u,v}\;\;\left(\mathrm{mod}\;q\right)\\ \; & =z_{u}+k_{u}+\sum_{v\in U_{3}\setminus C,u>v}k_{u,v}-\sum_{v\in U_{3}\setminus C,u<v}k_{u,v}+\sum_{v\in U_{2}\setminus \left(U_{3}\setminus C\right),u>v}k_{u,v}-\sum_{v\in U_{2}\setminus \left(U_{3}\setminus C\right),u<v}k_{u,v}\;\;\left(\mathrm{mod}\;q\right) \end{array}$$

user *u* computes

$$y_{u}=w_{u}\cdot t_{u}\cdot \prod_{v\in U_{2}\setminus \left(U_{3}\setminus C\right),u>v}t_{u,v}\cdot \prod_{v\in U_{2}\setminus \left(U_{3}\setminus C\right),u<v}t_{u,v}^{-1}\;\;\left(\mathrm{mod}\;R\right)$$

$$l_{u}=d_{u}+k_{u}+\sum_{v\in U_{2}\setminus \left(U_{3}\setminus C\right),u>v}k_{u,v}-\sum_{v\in U_{2}\setminus \left(U_{3}\setminus C\right),u<v}k_{u,v}\;\;\left(\mathrm{mod}\;q\right)$$

where ${\left\{w_{u}\right\}}_{u\in U_{3}\setminus C}\subset {\left(Z_{R}^{*}\right)}^{m}$ are uniform and random vectors satisfying $\prod_{u\in U_{3}\setminus C}w_{u}=\prod_{u\in U_{3}\setminus C}{\tilde{x}}_{u}$, ${\left\{d_{u}\right\}}_{u\in U_{3}\setminus C}\subset Z_{q}^{m}$ are uniform and random vectors satisfying $\sum_{u\in U_{3}\setminus C}d_{u}=\sum_{u\in U_{3}\setminus C}z_{u}$. Here $\prod_{u\in U_{3}\setminus C}{\tilde{x}}_{u}$ and $\sum_{u\in U_{3}\setminus C}z_{u}$ can be computed from the server’s output *X* and semi-honest users’ input vectors. For $X=(X^{1},\ldots ,X^{m})$, if $X^{i}\neq 0\left(1\leq i\leq m\right)$, let $Z^{i}=0$, let ${\tilde{X}}^{i}=X^{i}$; if $X^{i}=0$, let $Z^{i}$ be a random element from $Z_{q}\setminus \left\{0\right\}$, and let ${\tilde{X}}^{i}$ be a random element from $Z_{R}^{*}$. We get two vectors, $Z=(Z^{1},\ldots ,Z^{m})$ and $\tilde{X}=\left({\tilde{X}}^{1},\ldots ,{\tilde{X}}^{m}\right)$; thus, can compute

$$\prod_{u\in U_{3}\setminus C}{\tilde{x}}_{u}=\tilde{X}\cdot \prod_{u\in C}{\tilde{x}}_{u}^{-1}\;\;\left(\mathrm{mod}\;R\right)$$

$$\sum_{u\in U_{3}\setminus C}z_{u}=Z-\sum_{u\in C}z_{u}\;\;\left(\mathrm{mod}\;q\right)$$

By Lemmas A1 and A2 in Appendix B, together with the definition of $z_{u}$ and ${\tilde{x}}_{u}$ for user *u* in Setup Round, this hybrid is indistinguishable from the previous one. Define the probabilistic polynomial-time simulator SIM to be the distribution in this hybrid; then, the output of the simulator is computationally indistinguishable from ${\mathrm{VIEW}}_{C}$. Consider the case in which $|U_{3}|<t$; the protocol aborts in Round 3, and the server has no outputs. The idea of proof is similar.

${\mathrm{Hyb}}_{0}$

This random variable is distributed exactly as ${\mathrm{VIEW}}_{C}$, the joint view of semi-honest parties C in a real execution of the protocol.

${\mathrm{Hyb}}_{1}$

Compare to ${\mathrm{Hyb}}_{0}$; in this hybrid, we change the behavior of simulated honest parties in the set $U_{2}\setminus C$. When any user $u\in U_{2}\setminus C$ sends encrypted secret shares $e_{u,v}$ to any user $v\in U_{2}\setminus C$, instead of using $\mathrm{KA}.\mathrm{agree}(c_{u}^{sk},c_{v}^{pk})$ to encrypt and decrypt, they use a uniformly random encryption key, $c_{u,v}=c_{v,u}$, sampled by the simulator. The security of Diffie–Hellman key agreement guarantees that this hybrid is indistinguishable from the previous one.

${\mathrm{Hyb}}_{2}$

Compare to ${\mathrm{Hyb}}_{1}$; in this hybrid, when any user $u\in U_{2}\setminus C$ sends encrypted secret shares to any user, $v\in U_{2}\setminus C$, instead of sending the encryptions of the shares of $s_{u}^{sk}$ and $b_{u}$ corresponding to *v*, user *u* sends the encryption of 0 (with appropriate length) to user *v* via server. Since the key remains same and only the contents of the ciphertexts have changed, the IND-CPA security of the authenticated encryption scheme guarantees that this hybrid is indistinguishable from the previous one.

${\mathrm{Hyb}}_{3}$

Define $U^{*}=U_{2}\setminus C$. Compare to ${\mathrm{Hyb}}_{2}$, in this hybrid, when any user $u\in U^{*}$ sends encrypted secret shares to any user v∈C, instead of sending the encryptions of the shares of $s_{u}^{sk}$ and $b_{u}$ corresponding to *v*, user *u* sends the encryption of the shares of $s_{u}^{sk}$ and 0 (using a different sharing polynomial to share 0 for every $u\in U^{*}$) corresponding to *v* to user *v* via server. Since the protocol aborts before Round 4, semi-honest parties cannot get the shares of $b_{u}$ corresponding to any honest party, so there are only |C∖{S}|<t shares of $b_{u}$ contained in all the messages that the simulator has sent and received. The security of secret sharing guarantees that this hybrid is indistinguishable from the previous one.

${\mathrm{Hyb}}_{4}$

Compare to ${\mathrm{Hyb}}_{3}$; in this hybrid, when any user $u\in U^{*}$ computes the self masks $t_{u}$ and $k_{u}$ in Round 3, instead of using pseudorandom generators to get $t_{u}={\mathrm{PRG}}_{1}\left(b_{u}\right)$ and $k_{u}={\mathrm{PRG}}_{2}\left(b_{u}\right)$, user *u* takes $t_{u}\leftarrow {\left(Z_{R}^{*}\right)}^{m}$ and $k_{u}\leftarrow Z_{q}^{m}$ (with appropriate length) sampled uniformly and randomly by the simulator as self masks. In this hybrid, we substitute the output of a PRG on a randomly generated seed with a uniformly value on the output space, the security of the pseudorandom generator guarantees that this hybrid is indistinguishable from the previous one.

${\mathrm{Hyb}}_{5}$

Compare to ${\mathrm{Hyb}}_{4}$; in this hybrid, when any user $u\in U^{*}$ computes the masked input vector in Round 3, instead of computing

$$y_{u}={\tilde{x}}_{u}\cdot t_{u}\cdot \prod_{v\in U_{2},u>v}t_{u,v}\cdot \prod_{v\in U_{2},u<v}t_{u,v}^{-1}\;\;\left(\mathrm{mod}\;R\right)$$

$$l_{u}=z_{u}+k_{u}+\sum_{v\in U_{2},u>v}k_{u,v}-\sum_{v\in U_{2},u<v}k_{u,v}\;\;\left(\mathrm{mod}\;q\right)$$

user *u* computes

$$y_{u}=t_{u}\cdot \prod_{v\in U_{2},u>v}t_{u,v}\cdot \prod_{v\in U_{2},u<v}t_{u,v}^{-1}\;\;\left(\mathrm{mod}\;R\right)$$

$$l_{u}=k_{u}+\sum_{v\in U_{2},u>v}k_{u,v}-\sum_{v\in U_{2},u<v}k_{u,v}\;\;\left(\mathrm{mod}\;q\right)$$

As $t_{u}$ and $k_{u}$ are uniform and random vectors, and then ${\tilde{x}}_{u}\cdot t_{u}$ are uniform and random on ${\left(Z_{R}^{*}\right)}^{m}$, $z_{u}+k_{u}$ are uniform and random on $Z_{q}^{m}$, so this hybrid is indistinguishable from the previous one. Define the probabilistic polynomial-time simulator SIM as the distribution in this hybrid, then the output of the simulator is computationally indistinguishable from ${\mathrm{VIEW}}_{C}$. □

## Appendix E. Proof of Theorem 5

**Proof.** We show that the server will obtain the correct value of sum at 4th step of Round 4 for each additive aggregation execution.

$$\begin{array}{cc} (3)\mathrm{RHS} & =\sum_{u\in U_{3}^{\left(j\right)}}y_{u,j}-\sum_{u\in U_{3}^{\left(j\right)}}t_{u,j}+\sum_{u\in U_{3}^{\left(j\right)},v\in U_{2}^{\left(j\right)}\setminus U_{3}^{\left(j\right)},v>u}t_{v,j,u}-\sum_{u\in U_{3}^{\left(j\right)},v\in U_{2}^{\left(j\right)}\setminus U_{3}^{\left(j\right)},v<u}t_{v,j,u}\;\;\left(\mathrm{mod}\;R\right)\\ \; & =(\sum_{u\in U_{3}^{\left(j\right)}}x_{u,j}+\sum_{u\in U_{3}^{\left(j\right)}}t_{u,j}+\sum_{u\in U_{3}^{\left(j\right)},v\in U_{2}^{\left(j\right)},u>v}t_{u,j,v}-\sum_{u\in U_{3}^{\left(j\right)},v\in U_{2}^{\left(j\right)},u<v}t_{u,j,v})\\ \; & \;-\sum_{u\in U_{3}^{\left(j\right)}}t_{u,j}+\sum_{u\in U_{3}^{\left(j\right)},v\in U_{2}^{\left(j\right)}\setminus U_{3}^{\left(j\right)},v>u}t_{v,j,u}-\sum_{u\in U_{3}^{\left(j\right)},v\in U_{2}^{\left(j\right)}\setminus U_{3}^{\left(j\right)},v<u}t_{v,j,u}\;\;\left(\mathrm{mod}\;R\right)\\ \; & =\sum_{u\in U_{3}^{\left(j\right)}}x_{u,j}+\left(\sum_{u\in U_{3}^{\left(j\right)},v\in U_{2}^{\left(j\right)},u>v}t_{u,j,v}-\sum_{u\in U_{3}^{\left(j\right)},v\in U_{2}^{\left(j\right)}\setminus U_{3}^{\left(j\right)},v<u}t_{v,j,u}\right)\\ \; & \;-\left(\sum_{u\in U_{3}^{\left(j\right)},v\in U_{2}^{\left(j\right)},u<v}t_{u,j,v}-\sum_{u\in U_{3}^{\left(j\right)},v\in U_{2}^{\left(j\right)}\setminus U_{3}^{\left(j\right)},v>u}t_{v,j,u}\right)\;\;\left(\mathrm{mod}\;R\right)\\ \; & =\sum_{u\in U_{3}^{\left(j\right)}}x_{u,j}+\sum_{u\in U_{3}^{\left(j\right)},v\in U_{3}^{\left(j\right)},u>v}t_{u,j,v}-\sum_{u\in U_{3}^{\left(j\right)},v\in U_{3}^{\left(j\right)},u<v}t_{u,j,v}\;\;\left(\mathrm{mod}\;R\right)\\ \; & =\sum_{u\in U_{3}^{\left(j\right)}}x_{u,j}\;\;\left(\mathrm{mod}\;R\right)=\left(3\right)\mathrm{LHS} \end{array}$$

Therefore, each secure additive aggregation execution has the correct output result. □

## Appendix F. Proof of Theorem 7

**Proof.** For simplicity, we only prove that the semi-honest security holds for *j*th secure additive aggregation, that is

$${\mathrm{VIEW}}_{C}\left({\left\{x_{u,j}\right\}}_{u\in U},U_{1},U_{2}^{\left(j\right)},U_{3}^{\left(j\right)},U_{4}^{\left(j\right)}\right)\equiv {\mathrm{SIM}}_{C}\left(x_{C},X_{j},U_{1},U_{2}^{\left(j\right)},U_{3}^{\left(j\right)},U_{4}^{\left(j\right)}\right)$$

Firstly, we discuss the case that $|U_{3}^{\left(j\right)}|\geq t$. We will define a simulator SIM and its outputs through a series of subsequent modification to the random variable ${\mathrm{VIEW}}_{C}$, so that any two subsequent random variables are computationally indistinguishable.

${\mathrm{Hyb}}_{0}$

This random variable is distributed exactly as ${\mathrm{VIEW}}_{C}$, the joint view of semi-honest parties C in a real execution of the *j*th additive aggregation protocol.

${\mathrm{Hyb}}_{1}$

Compare to ${\mathrm{Hyb}}_{0}$; in this hybrid, we change the behavior of simulated honest parties in the set $U_{2}^{\left(j\right)}\setminus C$. When any user $u\in U_{2}^{\left(j\right)}\setminus C$ sends encrypted secret shares $e_{u,j,v}$ to any user $v\in U_{2}^{\left(j\right)}\setminus C$, and also when *v* decrypts this ciphertext (if they are still online in Round 4 of *j*th secure additive aggregation), instead of using $\mathrm{KA}.\mathrm{agree}(c_{u}^{sk},c_{v}^{pk})$ to encrypt and decrypt, they use a uniformly random encryption key, $c_{u,v}=c_{v,u}$, sampled by the simulator. The security of Diffie–Hellman key agreement guarantees that this hybrid is indistinguishable from the previous one.

${\mathrm{Hyb}}_{2}$

Compare to ${\mathrm{Hyb}}_{1}$; in this hybrid, when any user $u\in U_{2}^{\left(j\right)}\setminus C$ sends encrypted secret shares to any user $v\in U_{2}^{\left(j\right)}\setminus C$, instead of sending the encryptions of the shares of $s_{u,j}^{sk}$ and $b_{u,j}$ corresponding to *v*, user *u* sends the encryption of 0 (with appropriate length) to user *v* via server. However, when *v* decrypts the ciphertext (if they are still online in Round 4 of *j*th secure additive aggregation), they continue to respond with the correct shares of $s_{u,j}^{sk}$ and $b_{u,j}$. Since the key remains the same, and only the contents of the ciphertexts have changed, the IND-CPA security of the authenticated encryption scheme guarantees that this hybrid is indistinguishable from the previous one.

${\mathrm{Hyb}}_{3}$

Define $U^{*}=\left(U_{2}^{\left(j\right)}\setminus U_{3}^{\left(j\right)}\right)\setminus C$. Compare to ${\mathrm{Hyb}}_{2}$; in this hybrid, when any user $u\in U^{*}$ sends encrypted secret shares to any user v∈C, instead of sending the encryptions of the shares of $s_{u,j}^{sk}$ and $b_{u,j}$ corresponding to *v*, user *u* sends the encryption of the shares of $s_{u,j}^{sk}$ and 0 (using a different sharing polynomial to share 0 for every $u\in U^{*}$) corresponding to *v* to user *v* via server. Since user $u\in U^{*}$ has dropped out before they have successfully sent messages associated with their private input to the server, according to the protocol, semi-honest parties cannot get the shares of $b_{u,j}$ corresponding to any honest party in Round 4 of *j*th secure additive aggregation. So, there are only |C∖{S}|<t shares of $b_{u,j}$ contained in all the messages that the simulator has sent and received. The security of secret sharing guarantees that this hybrid is indistinguishable from the previous one.

${\mathrm{Hyb}}_{4}$

Compare to ${\mathrm{Hyb}}_{3}$; in this hybrid, when any user $u\in U_{3}^{\left(j\right)}\setminus C$ sends encrypted secret shares to any user v∈C, instead of sending the encryptions of the shares of $s_{u,j}^{sk}$ and $b_{u,j}$ corresponding to *v*, user *u* sends the encryption of the shares of 0 (using a different sharing polynomial to share 0 for every $u\in U_{3}^{\left(j\right)}\setminus C$) and $b_{u,j}$ corresponding to *v* to user *v* via server. Since user $u\in U_{3}^{\left(j\right)}\setminus C$ has successfully sent messages associated with their private input to the server in *j*th additive aggregation, according to the protocol, semi-honest parties cannot get the shares of $s_{u,j}^{sk}$ corresponding to any honest party in Round 4 of *j*th secure additive aggregation. So, there are only |C∖{S}|<t shares of $s_{u,j}^{sk}$ contained in all the messages that the simulator has sent and received. The security of secret sharing guarantees that this hybrid is indistinguishable from the previous one.

${\mathrm{Hyb}}_{5}$

Compare to ${\mathrm{Hyb}}_{4}$; in this hybrid, when any user $u\in U_{3}^{\left(j\right)}\setminus C$ computes the seed $s_{u,j,v}$ corresponding to user $v\in U_{3}^{\left(j\right)}\setminus C$ in Round 3 of the *j*th additive aggregation, instead of using $e\left(s_{u,j}^{sk},s_{v}^{pk}\right)=e\left(H{\left(j\right)}^{a_{u}},g^{a_{v}}\right)=e{\left(H\left(j\right),g\right)}^{a_{u}a_{v}}$ as the seed of cancellable masks, user *u* expands $s_{u,j,v}=s_{v,j,u}\in G$, which is uniformly random sampled by the simulator to get mask $t_{u,j,v}$. The security of Diffie–Hellman key agreement and the property of bilinear map guarantee that this hybrid is indistinguishable from the previous one.

${\mathrm{Hyb}}_{6}$

Compare to ${\mathrm{Hyb}}_{5}$; in this hybrid, when any user $u\in U_{3}^{\left(j\right)}\setminus C$ computes the mask $t_{u,j,v}$ corresponding to user $v\in U_{3}^{\left(j\right)}\setminus C$ in Round 3 of the *j*th additive aggregation, instead of using pseudorandom generators to get $t_{u,j,v}={\mathrm{PRG}}_{1}\left(s_{u,j,v}\right)$, user *u* takes $t_{u,j,v}=t_{v,j,u}\leftarrow Z_{R}^{m}$ (with appropriate length), which is sampled uniformly and randomly by the simulator as mask. In this hybrid, we substitute the output of a PRG on a randomly generated seed with a uniformly value on the output space; the security of pseudorandom generator guarantees that this hybrid is indistinguishable from the previous one.

${\mathrm{Hyb}}_{7}$

Compare to ${\mathrm{Hyb}}_{6}$; in this hybrid, when any user $u\in U_{3}^{\left(j\right)}\setminus C$ computes the masked input vector in Round 3 of *j*th secure additive aggregation, instead of computing

$$\begin{array}{cc} y_{u,j} & =x_{u,j}+t_{u,j}+\sum_{v\in U_{2}^{\left(j\right)},u>v}t_{u,j,v}-\sum_{v\in U_{2}^{\left(j\right)},u<v}t_{u,j,v}\;\;\left(\mathrm{mod}\;R\right)\\ \; & =x_{u,j}+t_{u,j}+\sum_{v\in U_{3}^{\left(j\right)}\setminus C,u>v}t_{u,j,v}-\sum_{v\in U_{3}^{\left(j\right)}\setminus C,u<v}t_{u,j,v}\\ \; & \;+\sum_{v\in U_{2}^{\left(j\right)}\setminus \left(U_{3}^{\left(j\right)}\setminus C\right),u>v}t_{u,j,v}-\sum_{v\in U_{2}^{\left(j\right)}\setminus \left(U_{3}^{\left(j\right)}\setminus C\right),u<v}t_{u,j,v}\;\;\left(\mathrm{mod}\;R\right) \end{array}$$

user *u* computes

$$y_{u,j}=w_{u,j}+t_{u,j}+\sum_{v\in U_{2}^{\left(j\right)}\setminus \left(U_{3}^{\left(j\right)}\setminus C\right),u>v}t_{u,j,v}-\sum_{v\in U_{2}^{\left(j\right)}\setminus \left(U_{3}^{\left(j\right)}\setminus C\right),u<v}t_{u,j,v}\;\;\left(\mathrm{mod}\;R\right)$$

where ${\left\{w_{u,j}\right\}}_{u\in U_{3}^{\left(j\right)}\setminus C}\subset Z_{R}^{m}$ are uniform and random vectors satisfying $\sum_{u\in U_{3}^{\left(j\right)}\setminus C}w_{u,j}=\sum_{u\in U_{3}^{\left(j\right)}\setminus C}x_{u,j}$. It is notable that $\sum_{u\in U_{3}^{\left(j\right)}\setminus C}x_{u,j}=X_{j}$ is exactly the output of the server. By Lemma A1 in Appendix B, this hybrid is indistinguishable from the previous one. Define the probabilistic polynomial-time simulator SIM as the distribution in this hybrid, then the output of the simulator is computationally indistinguishable from ${\mathrm{VIEW}}_{C}$. Consider the case in which $|U_{3}^{\left(j\right)}|<t$, the *j*th secure additive aggregation protocol aborts in Round 3, and the server has no output. The idea of the proof is similar.

${\mathrm{Hyb}}_{0}$

This random variable is distributed exactly as ${\mathrm{VIEW}}_{C}$, the joint view of semi-honest parties C in a real execution of the protocol of the *j*th additive aggregation protocol.

${\mathrm{Hyb}}_{1}$

Compare to ${\mathrm{Hyb}}_{0}$; in this hybrid, we change the behavior of simulated honest parties in the set $U_{2}^{\left(j\right)}\setminus C$. When any user $u\in U_{2}^{\left(j\right)}\setminus C$ sends encrypted secret shares $e_{u,j,v}$ to any user $v\in U_{2}^{\left(j\right)}\setminus C$, instead of using $\mathrm{KA}.\mathrm{agree}(c_{u}^{sk},c_{v}^{pk})$ to encrypt and decrypt, they use a uniformly random encryption key $c_{u,v}=c_{v,u}$ sampled by the simulator. The security of Diffie–Hellman key agreement guarantees that this hybrid is indistinguishable from the previous one.

${\mathrm{Hyb}}_{2}$

Compare to ${\mathrm{Hyb}}_{1}$; in this hybrid, when any user $u\in U_{2}^{\left(j\right)}\setminus C$ sends encrypted secret shares to any user $v\in U_{2}^{\left(j\right)}\setminus C$, instead of sending the encryptions of the shares of $s_{u,j}^{sk}$ and $b_{u,j}$ corresponding to *v*, user *u* sends the encryption of 0 (with appropriate length) to user *v* via server. Since the key remains the same, and only the contents of the ciphertexts have changed, the IND-CPA security of the authenticated encryption scheme guarantees that this hybrid is indistinguishable from the previous one.

${\mathrm{Hyb}}_{3}$

Define $U^{*}=U_{2}^{\left(j\right)}\setminus C$. Compare to ${\mathrm{Hyb}}_{2}$; in this hybrid, when any user $u\in U^{*}$ sends encrypted secret shares to any user v∈C, instead of sending the encryptions of the shares of $s_{u,j}^{sk}$ and $b_{u,j}$ corresponding to *v*, user *u* sends the encryption of the shares of $s_{u,j}^{sk}$ and 0 (using a different sharing polynomial to share 0 for every $u\in U^{*}$) corresponding to *v* to user *v* via server. Since the protocol aborts before Round 4 in the *j*th execution, semi-honest parties cannot get the shares of $b_{u,j}$ corresponding to any honest party, so there are only |C∖{S}|<t shares of $b_{u,j}$ contained in all the messages that the simulator has sent and received. The security of secret sharing guarantees that this hybrid is indistinguishable from the previous one.

${\mathrm{Hyb}}_{4}$

Compare to ${\mathrm{Hyb}}_{3}$; in this hybrid, when any user $u\in U^{*}$ computes the self mask $t_{u,j}$ in Round 3, instead of using pseudorandom generators to get $t_{u,j}={\mathrm{PRG}}_{2}\left(g^{b_{u,j}}\right)$, user *u* takes $t_{u,j}\leftarrow Z_{R}^{m}$ (with appropriate length), which is sampled uniformly and randomly by the simulator as a self mask. In this hybrid, we substitute the output of a PRG on a randomly generated seed with a uniform value on the output space; the security of pseudorandom generator guarantees that this hybrid is indistinguishable from the previous one.

${\mathrm{Hyb}}_{5}$

Compare to ${\mathrm{Hyb}}_{4}$; in this hybrid, when any user $u\in U^{*}$ computes the masked input vector in Round 3 of the *j*th secure additive aggregation, instead of computing

$$y_{u,j}=x_{u,j}+t_{u,j}+\sum_{v\in U_{2}^{\left(j\right)},u>v}t_{u,j,v}-\sum_{v\in U_{2}^{\left(j\right)},u<v}t_{u,j,v}\;\;\left(\mathrm{mod}\;R\right)$$

user *u* computes

$$y_{u,j}=t_{u,j}+\sum_{v\in U_{2}^{\left(j\right)},u>v}t_{u,j,v}-\sum_{v\in U_{2}^{\left(j\right)},u<v}t_{u,j,v}\;\;\left(\mathrm{mod}\;R\right)$$

As $t_{u,j}$ is a uniform and random vector, $x_{u,j}+t_{u,j}$ are uniform and random on $Z_{R}^{m}$, so this hybrid is indistinguishable from the previous one. Define the probabilistic polynomial-time simulator SIM as the distribution in this hybrid, and then the output of the simulator is computationally indistinguishable from ${\mathrm{VIEW}}_{C}$. □

## Appendix G. Key Reusable Secure Multiplicative Aggregation

A complete description of the key reusable secure multiplicative aggregation protocol is as follows.

**Theorem** **A1** (Correctness of the protocol $\Pi_{\mathrm{ReMulAgg}}$)**.**
*In the protocol $\Pi_{\mathrm{ReMulAgg}}$, for each multiplicative aggregation execution, if it has an output, then the output will be equal to the correct component-wise product of private input vectors with probability $\geq 1-\frac{1}{2^{\eta }}$.*

**Proof.** First, show that the server has obtained the correct value of sum and product at the 4th step of Round 4 for each multiplicative aggregation execution.

$$\begin{array}{cc} (A1)\mathrm{RHS} & =\sum_{u\in U_{3}^{\left(j\right)}}l_{u,j}-\sum_{u\in U_{3}^{\left(j\right)}}k_{u,j}+\sum_{u\in U_{3}^{\left(j\right)},v\in U_{2}^{\left(j\right)}\setminus U_{3}^{\left(j\right)},v>u}k_{v,j,u}-\sum_{u\in U_{3}^{\left(j\right)},v\in U_{2}^{\left(j\right)}\setminus U_{3}^{\left(j\right)},v<u}k_{v,j,u}\;\;\left(\mathrm{mod}\;q\right)\\ \; & =(\sum_{u\in U_{3}^{\left(j\right)}}z_{u,j}+\sum_{u\in U_{3}^{\left(j\right)}}k_{u,j}+\sum_{u\in U_{3}^{\left(j\right)},v\in U_{2}^{\left(j\right)},u>v}k_{u,j,v}-\sum_{u\in U_{3}^{\left(j\right)},v\in U_{2}^{\left(j\right)},u<v}k_{u,j,v})\\ \; & \;-\sum_{u\in U_{3}^{\left(j\right)}}k_{u,j}+\sum_{u\in U_{3}^{\left(j\right)},v\in U_{2}^{\left(j\right)}\setminus U_{3}^{\left(j\right)},v>u}k_{v,j,u}-\sum_{u\in U_{3}^{\left(j\right)},v\in U_{2}^{\left(j\right)}\setminus U_{3}^{\left(j\right)},v<u}k_{v,j,u}\;\;\left(\mathrm{mod}\;q\right)\\ \; & =\sum_{u\in U_{3}^{\left(j\right)}}z_{u,j}+\left(\sum_{u\in U_{3}^{\left(j\right)},v\in U_{2}^{\left(j\right)},u>v}k_{u,j,v}-\sum_{u\in U_{3}^{\left(j\right)},v\in U_{2}^{\left(j\right)}\setminus U_{3}^{\left(j\right)},v<u}k_{v,j,u}\right)\\ \; & \;-\left(\sum_{u\in U_{3}^{\left(j\right)},v\in U_{2}^{\left(j\right)},u<v}k_{u,j,v}-\sum_{u\in U_{3}^{\left(j\right)},v\in U_{2}^{\left(j\right)}\setminus U_{3}^{\left(j\right)},v>u}k_{v,j,u}\right)\;\;\left(\mathrm{mod}\;q\right)\\ \; & =\sum_{u\in U_{3}^{\left(j\right)}}z_{u,j}+\sum_{u\in U_{3}^{\left(j\right)},v\in U_{3}^{\left(j\right)},u>v}k_{u,j,v}-\sum_{u\in U_{3}^{\left(j\right)},v\in U_{3}^{\left(j\right)},u<v}k_{u,j,v}\;\;\left(\mathrm{mod}\;q\right)\\ \; & =\sum_{u\in U_{3}^{\left(j\right)}}z_{u,j}\;\;\left(\mathrm{mod}\;q\right)=\left(A1\right)\mathrm{LHS} \end{array}$$

It is similar to show that (A2) holds. So, the server obtains $\sum_{u\in U_{3}^{\left(j\right)}}z_{u,j}$ and $\prod_{u\in U_{3}^{\left(j\right)}}{\tilde{x}}_{u,j}$ correctly. Consider the set

$$T_{j}=\left\{i\in \left[m\right]|x_{u,j}^{i}\neq 0\;\mathrm{holds}\mathrm{for}\mathrm{all}\;u\in U_{3}^{\left(j\right)}\right\}$$

due to the definition of $z_{u,j}$ and ${\tilde{x}}_{u,j}$ in the Round 3 for *j*th secure multiplicative aggregation, it holds that $z_{u,j}^{i}=0$ for every $i\in T_{j},u\in U_{3}^{\left(j\right)}$; that is, $I_{j}\cap T_{j}=⌀$. It also holds that $x_{u,j}^{i}={\tilde{x}}_{u,j}^{i}$ for every $i\in T_{j},u\in U_{3}^{\left(j\right)}$. Multiply for all $u\in U_{3}^{\left(j\right)}$, and we get

$$\prod_{u\in U_{3}^{\left(j\right)}}x_{u,j}^{i}=\prod_{u\in U_{3}^{\left(j\right)}}{\tilde{x}}_{u,j}^{i}={\tilde{X}}_{j}^{i}=X_{j}^{i}\;\;\left(\mathrm{mod}\;R\right)$$

So, the *i*th $\left(\forall i\in T_{j}\right)$ component of the output vector in the *j*th secure multiplicative aggregation is the correct product result. Note that $\prod_{u\in U_{3}^{\left(j\right)}}x_{u,j}^{i}=0$ holds for every $i\in \left[m\right]\setminus T_{j}$; therefore, if the output is incorrect, the only case that may happen is that the *i*th component of the output vector is nonzero for some $i\in \left[m\right]\setminus T_{j}$. For any fixed $i\in \left[m\right]\setminus T_{j}$, consider the set

$$S_{i}^{\left(j\right)}=\left\{u\in U_{3}^{\left(j\right)}|x_{u,j}^{i}=0\right\}$$

Every user in this set randomly chooses an element from $Z_{q}\setminus \left\{0\right\}$ as $z_{u,j}^{i}$; these selections are independent of each other. Define the event $E_{i}^{\left(j\right)}$ as $\sum_{u\in U_{3}^{\left(j\right)}}z_{u,j}^{i}=\sum_{u\in S_{i}^{\left(j\right)}}z_{u,j}^{i}=0\;\;\left(\mathrm{mod}\;q\right)$, and then this event will happen with probability $\frac{1}{q-1}$. If event $E_{i}^{\left(j\right)}$ does not happen, then we have $i\in I_{j}$ by the protocol definition, so $X_{j}^{i}=0$, the *i*th component of the output vector is correct. The probability $P_{error}^{\left(j\right)}$ that the protocol outputs incorrectly in the *j*th secure multiplication is exactly the probability that event $E_{i}^{\left(j\right)}$ happens for some $i\in \left[m\right]\setminus T_{j}$. By Boole’s inequality

$$\begin{array}{cc} P_{error}^{\left(j\right)} & =\mathrm{Pr}[\exists i\in \left[m\right]\setminus T_{j}\;\mathrm{such}\mathrm{that}\;E_{i}^{\left(j\right)}\;\mathrm{happens}]\\ \; & \leq \sum_{i\in \left[m\right]\setminus T_{j}}\mathrm{Pr}\left[E_{i}^{\left(j\right)}\right]\\ \; & \leq \frac{m}{q-1}<\frac{1}{2^{\eta }} \end{array}$$

So, each secure multiplicative aggregation execution has the correct output result with probability of at least $1-\frac{1}{2^{\eta }}$. □

Next, we talk about the security of the protocol $\Pi_{\mathrm{ReMulAgg}}$. We will prove that the protocol is secure against static semi-honest adversaries, no matter when the users drop out during the protocol execution.

In the key reusable secure multiplicative aggregation protocol, there is a central server, S, a set of initial users *U* with |U|=n, and a threshold parameter, *t*. Users are allowed to drop out at any time of the entire protocol and to rejoin the protocol for a new multiplicative aggregation execution; denote the set of users whose message has been received by the server in Round *i* of *j*th secure multiplicative aggregation as $U_{i}^{\left(j\right)}$. We have $U⊇U_{1}⊇U_{2}^{\left(j\right)}⊇U_{3}^{\left(j\right)}⊇U_{4}^{\left(j\right)}$. Denote $U_{r}\left(r=2,3,4\right)$ as the set composed of $U_{r}^{\left(j\right)}$ for all *j*. Each user *u* has a private input vector, $x_{u,j}$, for the *j*th secure multiplication; denote $x_{U^{\prime }}={\left\{x_{u,j}\;\mathrm{for}\mathrm{all}\;j\right\}}_{u\in U^{\prime }}$, where $U^{\prime }$ can be any subset of users. The same as the definition in model statement, ${\mathrm{VIEW}}_{u}\left(x_{U}\right)$ represents the view of party u∈U∪{S} when the protocol runs with inputs $x_{U}$. Define C⊂U∪{S} as the set of semi-honest parties, and then ${\mathrm{VIEW}}_{C}\left(x_{U},U_{1},U_{2},U_{3},U_{4}\right)$ is the joint view of semi-honest adversaries during the extire protocol.

Firstly, we will consider the case in which semi-honest parties are a set of users, that is, C⊂U.

**Theorem** **A2** (Security of the protocol $\Pi_{\mathrm{ReMulAgg}}$ against semi-honest users)**.**
*There exists a probabilistic polynomial-time simulator, SIM, such that, for any security parameter λ, threshold parameter t, set of users U with |U|≥t, input vectors $x_{U}$, sets of online users $U⊇U_{1}⊇U_{2}^{\left(j\right)}⊇U_{3}^{\left(j\right)}⊇U_{4}^{\left(j\right)}$ for all j and set of semi-honest parties C⊆U with |C|≤t, the output of SIM is perfectly indistinguishable from ${\mathrm{VIEW}}_{C}$ as random variables, that is*

$${\mathrm{VIEW}}_{C}\left(x_{U},U_{1},U_{2},U_{3},U_{4}\right)\equiv {\mathrm{SIM}}_{C}\left(x_{C},U_{1},U_{2},U_{3},U_{4}\right)$$

**Proof.** For any user u∈C, all they have received during the entire protocol execution are public keys ${\left\{c_{v}^{pk},s_{v}^{pk}\right\}}_{v\in U_{1}\setminus \left\{u\right\}}$, ciphertexts ${\left\{e_{v,j,u}\right\}}_{v\in U_{2}^{\left(j\right)}\setminus \left\{u\right\}}$ for all *j* and a set of $U_{3}={\left\{U_{3}^{\left(j\right)}\right\}}_{j}$. Note that these messages do not depend on the inputs of the honest users. In fact, the only values sent by each honest user *v* that depend on his private input are $y_{v,j}$ and $l_{v,j}$, which they sent to the server in Round 3 of the *j*th execution, but the server is honest. So, when knowing the inputs $x_{C}$ of user set C, the simulator SIM can be defined to use zero vectors for the inputs of all honest users and to use $x_{u,j}\left(u\in C\right)$ for the inputs of semi-honest users *u* in the *j*th multiplicative aggregation and then to simulate the execution of the *j*th secure multiplicative aggregation protocol. The simulator outputs the joint view of users in C in the simulation process, which is perfectly indistinguishable from ${\mathrm{VIEW}}_{C}$. □

When the server is semi-honest, it can share what it has received in the protocol execution with other semi-honest users. We show that the view of any such group of semi-honest parties can be simulated by a simulator, which is given the inputs of the users in that group and the final outputs of the server.

**Theorem** **A3** (Security of the protocol $\Pi_{\mathrm{ReMulAgg}}$ against semi-honest server and users)**.**
*There exists a probability polynomial-time simulator, SIM, such that, for any security parameter λ, threshold parameter t, set of users U with |U|≥t, input vectors $x_{U}$, sets of online users $U⊇U_{1}⊇U_{2}^{\left(j\right)}⊇U_{3}^{\left(j\right)}⊇U_{4}^{\left(j\right)}$ for all j and set of semi-honest parties C⊆U∪{S} with |C∖{S}|<t, the output of SIM is perfectly indistinguishable from ${\mathrm{VIEW}}_{C}$ as random variables; that is,*

$${\mathrm{VIEW}}_{C}\left(x_{U},U_{1},U_{2},U_{3},U_{4}\right)\equiv {\mathrm{SIM}}_{C}\left(x_{C},X={\left\{X_{j}\right\}}_{j},U_{1},U_{2},U_{3},U_{4}\right)$$

*where*

$$X_{j}=\left\{\begin{array}{c} \left(X_{j}^{1},\ldots ,X_{j}^{m}\right)\;\;\;\;\;\;\;\mathrm{if}\;\left|U_{3}^{\left(j\right)}\right|\geq \;t\\ ⊥\;\;\;\;\;\;\;\;\;\;\;\;\;\;\;\;\;\;\;\;\mathrm{otherwise} \end{array}\right.$$

The proof of the theorem is based on the security of Diffie–Hellman key agreement, the IND-CPA security of the authenticated encryption scheme, the security of secret sharing, the security of the pseudorandom generator and the property of bilinear map.

**Proof.** For simplicity, we only prove that the semi-honest security holds for the *j*th secure multiplicative aggregation, that is

$${\mathrm{VIEW}}_{C}\left({\left\{x_{u,j}\right\}}_{u\in U},U_{1},U_{2}^{\left(j\right)},U_{3}^{\left(j\right)},U_{4}^{\left(j\right)}\right)\equiv {\mathrm{SIM}}_{C}\left(x_{C},X_{j},U_{1},U_{2}^{\left(j\right)},U_{3}^{\left(j\right)},U_{4}^{\left(j\right)}\right)$$

Firstly, we discuss the case in which $|U_{3}^{\left(j\right)}|\geq t$. We will define a simulator, SIM, and its outputs through a series of subsequent modifications to the random variable ${\mathrm{VIEW}}_{C}$, so that any two subsequent random variables are computationally indistinguishable.

${\mathrm{Hyb}}_{0}$

This random variable is distributed exactly as ${\mathrm{VIEW}}_{C}$, the joint view of semi-honest parties C in a real execution of the *j*th multiplicative aggregation protocol.

${\mathrm{Hyb}}_{1}$

Compare to ${\mathrm{Hyb}}_{0}$; in this hybrid, we change the behavior of simulated honest parties in the set $U_{2}^{\left(j\right)}\setminus C$. When any user $u\in U_{2}^{\left(j\right)}\setminus C$ sends encrypted secret shares $e_{u,j,v}$ to any user $v\in U_{2}^{\left(j\right)}\setminus C$, and also when *v* decrypts this ciphertext (if they are still online in Round 4 of the *j*th secure multiplicative aggregation), instead of using $\mathrm{KA}.\mathrm{agree}(c_{u}^{sk},c_{v}^{pk})$ to encrypt and decrypt, they use a uniformly random encryption key, $c_{u,v}=c_{v,u}$, sampled by the simulator. The security of Diffie–Hellman key agreement guarantees that this hybrid is indistinguishable from the previous one.

${\mathrm{Hyb}}_{2}$

Compare to ${\mathrm{Hyb}}_{1}$; in this hybrid, when any user $u\in U_{2}^{\left(j\right)}\setminus C$ sends encrypted secret shares to any user $v\in U_{2}^{\left(j\right)}\setminus C$, instead of sending the encryptions of the shares of $s_{u,j}^{sk}$ and $b_{u,j}$ corresponding to *v*, user *u* sends the encryption of 0 (with appropriate length) to user *v* via server. However, when *v* decrypts the ciphertext (if they are still online in Round 4 of *j*th secure multiplicative aggregation), they continue to respond with the correct shares of $s_{u,j}^{sk}$ and $b_{u,j}$. Since the key remains the same, and only the contents of the ciphertexts have changed, the IND-CPA security of the authenticated encryption scheme guarantees that this hybrid is indistinguishable from the previous one.

${\mathrm{Hyb}}_{3}$

Define $U^{*}=\left(U_{2}^{\left(j\right)}\setminus U_{3}^{\left(j\right)}\right)\setminus C$. Compare to ${\mathrm{Hyb}}_{2}$; in this hybrid, when any user $u\in U^{*}$ sends encrypted secret shares to any user v∈C, instead of sending the encryptions of the shares of $s_{u,j}^{sk}$ and $b_{u,j}$ corresponding to *v*, user *u* sends the encryption of the shares of $s_{u,j}^{sk}$ and 0 (using a different sharing polynomial to share 0 for every $u\in U^{*}$) corresponding to *v* to user *v* via server. Since user $u\in U^{*}$ has dropped out before they have successfully sent messages associated with their private input to the server, according to the protocol, semi-honest parties cannot obtain the shares of $b_{u,j}$ corresponding to any honest party in Round 4 of the *j*th secure multiplicative aggregation. So, there are only |C∖{S}|<t shares of $b_{u,j}$ contained in all the messages that the simulator has sent and received. The security of secret sharing guarantees that this hybrid is indistinguishable from the previous one.

${\mathrm{Hyb}}_{4}$

Compare to ${\mathrm{Hyb}}_{3}$; in this hybrid, when any user $u\in U_{3}^{\left(j\right)}\setminus C$ sends encrypted secret shares to any user v∈C, instead of sending the encryptions of the shares of $s_{u,j}^{sk}$ and $b_{u,j}$ corresponding to *v*, user *u* sends the encryption of the shares of 0 (using a different sharing polynomial to share 0 for every $u\in U_{3}^{\left(j\right)}\setminus C$) and $b_{u,j}$ corresponding to *v* to user *v* via server. Since user $u\in U_{3}^{\left(j\right)}\setminus C$ has successfully sent messages associated with their private input to the server in the *j*th multiplication, according to the protocol, semi-honest parties cannot get the shares of $s_{u,j}^{sk}$ corresponding to any honest party in Round 4 of *j*th secure multiplicative aggregation. So, there are only |C∖{S}|<t shares of $s_{u,j}^{sk}$ contained in all the messages that the simulator has sent and received. The security of secret sharing guarantees that this hybrid is indistinguishable from the previous one.

${\mathrm{Hyb}}_{5}$

Compare to ${\mathrm{Hyb}}_{4}$; in this hybrid, when any user $u\in U_{3}^{\left(j\right)}\setminus C$ computes the seed $s_{u,j,v}$ corresponding to user $v\in U_{3}^{\left(j\right)}\setminus C$ in Round 3 of the *j*th multiplicative aggregation, instead of using $e\left(s_{u,j}^{sk},s_{v}^{pk}\right)=e\left(H{\left(j\right)}^{a_{u}},g^{a_{v}}\right)=e{\left(H\left(j\right),g\right)}^{a_{u}a_{v}}$ as the seed of cancellable masks, user *u* expands $s_{u,j,v}=s_{v,j,u}\in G$, which is uniformly random sampled by the simulator to obtain masks $t_{u,j,v}$ and $k_{u,j,v}$. The security of Diffie–Hellman key agreement and the property of the bilinear map guarantee that this hybrid is indistinguishable from the previous one.

${\mathrm{Hyb}}_{6}$

Compare to ${\mathrm{Hyb}}_{5}$; in this hybrid, when any user $u\in U_{3}^{\left(j\right)}\setminus C$ computes the masks $t_{u,j,v}$ and $k_{u,j,v}$ corresponding to user $v\in U_{3}^{\left(j\right)}\setminus C$ in Round 3 of the *j*th multiplicative aggregation, instead of using pseudorandom generators to get $t_{u,j,v}={\mathrm{PRG}}_{1}\left(s_{u,j,v}\right)$ and $k_{u,j,v}={\mathrm{PRG}}_{2}\left(s_{u,j,v}\right)$, user *u* takes $t_{u,j,v}=t_{v,j,u}\leftarrow {\left(Z_{R}^{*}\right)}^{m}$ and $k_{u,j,v}=k_{v,j,u}\leftarrow Z_{q}^{m}$ (with appropriate length), which are sampled uniformly and randomly by the simulator as masks. In this hybrid, we substitute the output of a PRG on a randomly generated seed with a uniform value on the output space; the security of pseudorandom generator guarantees that this hybrid is indistinguishable from the previous one.

${\mathrm{Hyb}}_{7}$

Compare to ${\mathrm{Hyb}}_{6}$, in this hybrid, when any user $u\in U_{3}^{\left(j\right)}\setminus C$ computes the masked input vector in Round 3 of the *j*th secure multiplicative aggregation, instead of computing

$$\begin{array}{cc} y_{u,j} & ={\tilde{x}}_{u,j}\cdot t_{u,j}\cdot \prod_{v\in U_{2}^{\left(j\right)},u>v}t_{u,j,v}\cdot \prod_{v\in U_{2}^{\left(j\right)},u<v}t_{u,j,v}^{-1}\;\;\left(\mathrm{mod}\;R\right)\\ \; & ={\tilde{x}}_{u,j}\cdot t_{u,j}\cdot \prod_{v\in U_{3}^{\left(j\right)}\setminus C,u>v}t_{u,j,v}\cdot \prod_{v\in U_{3}^{\left(j\right)}\setminus C,u<v}t_{u,j,v}^{-1}\\ \; & \;\cdot \prod_{v\in U_{2}^{\left(j\right)}\setminus \left(U_{3}^{\left(j\right)}\setminus C\right),u>v}t_{u,j,v}\cdot \prod_{v\in U_{2}^{\left(j\right)}\setminus \left(U_{3}^{\left(j\right)}\setminus C\right),u<v}t_{u,j,v}^{-1}\;\;\left(\mathrm{mod}\;R\right) \end{array}$$

$$\begin{array}{cc} l_{u,j} & =z_{u,j}+k_{u,j}+\sum_{v\in U_{2}^{\left(j\right)},u>v}k_{u,j,v}-\sum_{v\in U_{2}^{\left(j\right)},u<v}k_{u,j,v}\;\;\left(\mathrm{mod}\;q\right)\\ \; & =z_{u,j}+k_{u,j}+\sum_{v\in U_{3}^{\left(j\right)}\setminus C,u>v}k_{u,j,v}-\sum_{v\in U_{3}^{\left(j\right)}\setminus C,u<v}k_{u,j,v}\\ \; & \;+\sum_{v\in U_{2}^{\left(j\right)}\setminus \left(U_{3}^{\left(j\right)}\setminus C\right),u>v}k_{u,j,v}-\sum_{v\in U_{2}^{\left(j\right)}\setminus \left(U_{3}^{\left(j\right)}\setminus C\right),u<v}k_{u,j,v}\;\;\left(\mathrm{mod}\;q\right) \end{array}$$

user *u* computes

$$y_{u,j}=w_{u,j}\cdot t_{u,j}\cdot \prod_{v\in U_{2}^{\left(j\right)}\setminus \left(U_{3}^{\left(j\right)}\setminus C\right),u>v}t_{u,j,v}\cdot \prod_{v\in U_{2}^{\left(j\right)}\setminus \left(U_{3}^{\left(j\right)}\setminus C\right),u<v}t_{u,j,v}^{-1}\;\;\left(\mathrm{mod}\;R\right)$$

$$l_{u,j}=d_{u,j}+k_{u,j}+\sum_{v\in U_{2}^{\left(j\right)}\setminus \left(U_{3}^{\left(j\right)}\setminus C\right),u>v}k_{u,j,v}-\sum_{v\in U_{2}^{\left(j\right)}\setminus \left(U_{3}^{\left(j\right)}\setminus C\right),u<v}k_{u,j,v}\;\;\left(\mathrm{mod}\;q\right)$$

where ${\left\{w_{u,j}\right\}}_{u\in U_{3}^{\left(j\right)}\setminus C}\subset {\left(Z_{R}^{*}\right)}^{m}$ are uniform and random vectors satisfying $\prod_{u\in U_{3}^{\left(j\right)}\setminus C}w_{u,j}=\prod_{u\in U_{3}^{\left(j\right)}\setminus C}{\tilde{x}}_{u,j}$, while ${\left\{d_{u,j}\right\}}_{u\in U_{3}^{\left(j\right)}\setminus C}\subset Z_{q}^{m}$ are uniform and random vectors satisfying $\sum_{u\in U_{3}^{\left(j\right)}\setminus C}d_{u,j}=\sum_{u\in U_{3}^{\left(j\right)}\setminus C}z_{u,j}$. It is notable that $\prod_{u\in U_{3}^{\left(j\right)}\setminus C}{\tilde{x}}_{u,j}$ and $\sum_{u\in U_{3}^{\left(j\right)}\setminus C}z_{u,j}$ can be computed from the server’s output $X_{j}$ and semi-honest users’ input vectors. For $X_{j}=\left(X_{j}^{1},\ldots ,X_{j}^{m}\right)$, if $X_{j}^{i}\neq 0\left(1\leq i\leq m\right)$, let $Z_{j}^{i}=0$, let ${\tilde{X}}_{j}^{i}=X_{j}^{i}$; if $X_{j}^{i}=0$, let $Z_{j}^{i}$ be a random element from $Z_{q}\setminus \left\{0\right\}$, and let ${\tilde{X}}_{j}^{i}$ be a random element from $Z_{R}^{*}$. We obtain two vectors, $Z_{j}=\left(Z_{j}^{1},\ldots ,Z_{j}^{m}\right)$ and ${\tilde{X}}_{j}=\left({\tilde{X}}_{j}^{1},\ldots ,{\tilde{X}}_{j}^{m}\right)$, and can thus compute

$$\prod_{u\in U_{3}^{\left(j\right)}\setminus C}{\tilde{x}}_{u,j}={\tilde{X}}_{j}\cdot \prod_{u\in C}{\tilde{x}}_{u,j}^{-1}\;\;\left(\mathrm{mod}\;R\right)$$

$$\sum_{u\in U_{3}^{\left(j\right)}\setminus C}z_{u,j}=Z_{j}-\sum_{u\in C}z_{u,j}\;\;\left(\mathrm{mod}\;q\right)$$

By Lemmas A1 and A2 in Appendix B, together with the definition of $z_{u,j}$ and ${\tilde{x}}_{u,j}$ for user *u* in Round 3 of *j*th secure multiplicative aggregation, this hybrid is indistinguishable from the previous one. Define the probabilistic polynomial-time simulator SIM to be the distribution in this hybrid, then the output of the simulator is computationally indistinguishable from ${\mathrm{VIEW}}_{C}$. Consider the case in which $|U_{3}^{\left(j\right)}|<t$, the *j*th secure multiplicative aggregation protocol aborts in Round 3, and the server has no output. The idea of the proof is similar.

${\mathrm{Hyb}}_{0}$

This random variable is distributed exactly as ${\mathrm{VIEW}}_{C}$, the joint view of semi-honest parties C in a real execution of the protocol of the *j*th multiplicative aggregation protocol.

${\mathrm{Hyb}}_{1}$

Compare to ${\mathrm{Hyb}}_{0}$; in this hybrid, we change the behavior of simulated honest parties in the set $U_{2}^{\left(j\right)}\setminus C$. When any user $u\in U_{2}^{\left(j\right)}\setminus C$ sends encrypted secret shares $e_{u,j,v}$ to any user $v\in U_{2}^{\left(j\right)}\setminus C$, instead of using $\mathrm{KA}.\mathrm{agree}(c_{u}^{sk},c_{v}^{pk})$ to encrypt and decrypt, they use a uniformly random encryption key $c_{u,v}=c_{v,u}$ sampled by the simulator. The security of Diffie–Hellman key agreement guarantees that this hybrid is indistinguishable from the previous one.

${\mathrm{Hyb}}_{2}$

Compare to ${\mathrm{Hyb}}_{1}$; in this hybrid, when any user $u\in U_{2}^{\left(j\right)}\setminus C$ sends encrypted secret shares to any user $v\in U_{2}^{\left(j\right)}\setminus C$, instead of sending the encryptions of the shares of $s_{u,j}^{sk}$ and $b_{u,j}$ corresponding to *v*, user *u* sends the encryption of 0 (with appropriate length) to user *v* via server. Since the key remains the same, and only the contents of the ciphertexts have changed, the IND-CPA security of the authenticated encryption scheme guarantees that this hybrid is indistinguishable from the previous one.

${\mathrm{Hyb}}_{3}$

Define $U^{*}=U_{2}^{\left(j\right)}\setminus C$. Compare to ${\mathrm{Hyb}}_{2}$, in this hybrid, when any user $u\in U^{*}$ sends encrypted secret shares to any user v∈C, instead of sending the encryptions of the shares of $s_{u,j}^{sk}$ and $b_{u,j}$ corresponding to *v*, user *u* sends the encryption of the shares of $s_{u,j}^{sk}$ and 0 (using a different sharing polynomial to share 0 for every $u\in U^{*}$) corresponding to *v* to user *v* via the server. Since the protocol aborts before Round 4 in *j*th execution, semi-honest parties cannot get the shares of $b_{u,j}$ corresponding to any honest party, so there are only |C∖{S}|<t shares of $b_{u,j}$ contained in all the messages that the simulator has sent and received. The security of secret sharing guarantees that this hybrid is indistinguishable from the previous one.

${\mathrm{Hyb}}_{4}$

Compare to ${\mathrm{Hyb}}_{3}$; in this hybrid, when any user $u\in U^{*}$ computes the self masks $t_{u,j}$ and $k_{u,j}$ in Round 3, instead of using pseudorandom generators to get $t_{u,j}={\mathrm{PRG}}_{3}\left(g^{b_{u,j}}\right)$ and $k_{u,j}={\mathrm{PRG}}_{4}\left(g^{b_{u,j}}\right)$, user *u* takes $t_{u,j}\leftarrow {\left(Z_{R}^{*}\right)}^{m}$ and $k_{u,j}\leftarrow Z_{q}^{m}$ (with appropriate length), which are sampled uniformly and randomly by the simulator as self masks. In this hybrid, we substitute the output of a PRG on a randomly generated seed with a uniform value on the output space; the security of the pseudorandom generator guarantees that this hybrid is indistinguishable from the previous one.

${\mathrm{Hyb}}_{5}$

Compare to ${\mathrm{Hyb}}_{4}$, in this hybrid, when any user $u\in U^{*}$ computes the masked input vector in Round 3 of the *j*th secure multiplicative aggregation; instead of computing

$$y_{u,j}={\tilde{x}}_{u,j}\cdot t_{u,j}\cdot \prod_{v\in U_{2}^{\left(j\right)},u>v}t_{u,j,v}\cdot \prod_{v\in U_{2}^{\left(j\right)},u<v}t_{u,j,v}^{-1}\;\;\left(\mathrm{mod}\;R\right)$$

$$l_{u,j}=z_{u,j}+k_{u,j}+\sum_{v\in U_{2}^{\left(j\right)},u>v}k_{u,j,v}-\sum_{v\in U_{2}^{\left(j\right)},u<v}k_{u,j,v}\;\;\left(\mathrm{mod}\;q\right)$$

user *u* computes

$$y_{u,j}=t_{u,j}\cdot \prod_{v\in U_{2}^{\left(j\right)},u>v}t_{u,j,v}\cdot \prod_{v\in U_{2}^{\left(j\right)},u<v}t_{u,j,v}^{-1}\;\;\left(\mathrm{mod}\;R\right)$$

$$l_{u,j}=k_{u,j}+\sum_{v\in U_{2}^{\left(j\right)},u>v}k_{u,j,v}-\sum_{v\in U_{2}^{\left(j\right)},u<v}k_{u,j,v}\;\;\left(\mathrm{mod}\;q\right)$$

As $t_{u,j}$ and $k_{u,j}$ are uniform and random vectors, ${\tilde{x}}_{u,j}\cdot t_{u,j}$ are uniform and random on ${\left(Z_{R}^{*}\right)}^{m}$, while $z_{u,j}+k_{u,j}$ are uniform and random on $Z_{q}^{m}$, so this hybrid is indistinguishable from the previous one. Define the probabilistic polynomial-time simulator SIM as the distribution in this hybrid, and then the output of the simulator is computationally indistinguishable from ${\mathrm{VIEW}}_{C}$. □

Finally, we show that the long-term keys of honest users remain safe during multiple aggregation executions. For the case where C⊆U with |C|<t, for any honest user u∈U, the adversary cannot reconstruct the one-time private key $s_{u,j}^{sk}$ for any *j* since the number of shares it holds is less than the secret sharing threshold *t*. Therefore, the adversary learns nothing about the long-term private key $s_{u}^{sk}$. For the case where C⊆U with |C|=t, for any honest user u∈U, the adversary can reconstruct the one-time private key $s_{u,j}^{sk}=H{\left(j\right)}^{s_{u}^{sk}}$ for any *j*. According to the hardness assumption of a discrete logarithm, the adversary learns nothing about the long-term private key $s_{u}^{sk}$. For the case where the server is semi-honest, that is, C⊆U∪{S} with |C∖{S}|<t, for any honest user u∈U, the adversary can learn the one-time private key $s_{u,j}^{sk}=H{\left(j\right)}^{s_{u}^{sk}}$ only when $u\in U_{2}^{\left(j\right)}\setminus U_{3}^{\left(j\right)}$. According to the hardness assumption of discrete logarithm, the adversary learns nothing about the long-term private key $s_{u}^{sk}$.
